# The role of kinesin-1 in neuronal dense core vesicle transport, locomotion and lifespan regulation in *C. elegans*

**DOI:** 10.1242/jcs.262148

**Published:** 2024-09-06

**Authors:** Anna Gavrilova, Astrid Boström, Nickolay Korabel, Sergei Fedotov, Gino B. Poulin, Victoria J. Allan

**Affiliations:** ^1^ School of Biological Sciences, Faculty of Biology, Medicine and Health, University of Manchester, The Michael Smith Building, Rumford St, Manchester M13 9PT, UK; ^2^ Department of Mathematics, Faculty of Science and Engineering, The University of Manchester, Manchester M13 9PL, UK

**Keywords:** Kinesin, Dense core vesicle, Neuron, *C. elegans*, Lifespan, Locomotion

## Abstract

Fast axonal transport is crucial for neuronal function and is driven by kinesins and cytoplasmic dynein. Here, we investigated the role of kinesin-1 in dense core vesicle (DCV) transport in *C. elegans*, using mutants in the kinesin light chains (*klc-1* and *klc*-2) and the motor subunit (*unc-116*) expressing an *ida-1::gfp* transgene that labels DCVs. DCV transport in both directions was greatly impaired in an *unc-116* mutant and had reduced velocity in a *klc-2* mutant. In contrast, the speed of retrograde DCV transport was increased in a *klc-1* mutant whereas anterograde transport was unaffected. We identified striking differences between the *klc* mutants in their effects on worm locomotion and responses to drugs affecting neuromuscular junction activity. We also determined lifespan, finding that *unc-116* mutant was short-lived whereas the *klc* single mutant lifespan was wild type. The *ida-1::gfp* transgenic strain was also short-lived, but surprisingly, *klc-1* and *klc-2* extended the *ida-1::gfp* lifespan beyond that of wild type. Our findings suggest that kinesin-1 not only influences anterograde and retrograde DCV transport but is also involved in regulating lifespan and locomotion, with the two kinesin light chains playing distinct roles.

## Teaser

**Summary:** Kinesin-1 and its light chains play distinct roles in dense core vesicle movement, worm locomotion and determining the lifespan of worms expressing a dense core vesicle marker.

## INTRODUCTION

Intracellular transport along microtubules drives the movement of organelles, vesicles and biomolecules over long distances within animal cells. It is crucial for maintaining cellular organisation, homeostasis and overall cell viability (Sleigh et al., 2019). This is particularly important in neurons, given that most proteins are synthesised and packaged in the cell body and must be effectively transported over substantial distances. A direct link has been observed between mutations in the microtubule motor proteins that drive this transport and a spectrum of nervous system disorders and neurological diseases (Brady and Morfini, 2017; Chiba and Niwa, 2024; Sleigh et al., 2019). Nonetheless, the relationship between disruptions in axonal transport and neuronal disorders is not fully understood.

Intracellular cargoes undergo active transport with the aid of two distinct ATP-dependent motor proteins: kinesins and dyneins (Cason and Holzbaur, 2022; Hirokawa et al., 2010). Cytoplasmic dynein drives retrograde transport, towards microtubule minus ends, whereas most kinesin family members support anterograde transport, towards microtubule plus ends. In axons, microtubules align with their plus-ends extending towards the axon tip, with the minus-ends pointing towards the cell body (Baas and Lin, 2011). More than one kinesin type can often transport the same cargo (e.g. Cason and Holzbaur, 2022; Guardia et al., 2016; Gumy et al., 2017; Kulkarni et al., 2017; Lim et al., 2017; Serra-Marques et al., 2020; Zahavi et al., 2021); however, the specific contributions and roles of each motor and how they work together is poorly understood.

Dense core vesicles (DCVs) are specialised membrane-bound organelles responsible for the transport of neuropeptides, monoamines, neurotrophic factors, hormones and insulin-related peptides to their sites of release in both axons and dendrites (Gondre-Lewis et al., 2012; Wong et al., 2012). This traffic is essential for neuronal growth, signalling, learning, development, movement and ageing (Gondre-Lewis et al., 2012; Randi et al., 2023; Ripoll-Sanchez et al., 2023). DCVs are formed in the neuron soma, undergo fast axonal transport and release their content in response to specific stimuli, with fusion occurring at both synaptic and non-synaptic sites (Bharat et al., 2017; Hammarlund et al., 2008; Schlager et al., 2010; Shakiryanova et al., 2006; Wong et al., 2012).

DCVs move in both directions in *C. elegans* (e.g. Goodwin et al., 2012; Zahn et al., 2004), *Drosophila* (e.g. Lim et al., 2017; Lund et al., 2021) and vertebrates (e.g. Arimura et al., 2009; Gumy et al., 2017; Kwinter et al., 2009; Schlager et al., 2010), with reversals being reported to occur primarily at axon terminals or synapses (Bharat et al., 2017; Cason and Holzbaur, 2022; Wong et al., 2012). Kinesin-3 (UNC-104 in worms) has been shown to drive plus-end-directed DCV movement, and kinesin-3 mutations or depletions greatly reduce the number of DCVs entering the axon (e.g. Barkus et al., 2008; Bharat et al., 2017; Goodwin et al., 2012; Gumy et al., 2017; Laurent et al., 2018; Lo et al., 2011; Park et al., 2023; Siddiqui et al., 2019; Zahn et al., 2004). Importantly, kinesin-1 is also a DCV motor in vertebrates and flies, leading to the hypothesis that kinesin-3 moves DCVs out of the cell body, then kinesin-1 takes over from, or works alongside, kinesin-3 for long distance movement (Arimura et al., 2009; Gumy et al., 2017; Lim et al., 2017; Zahavi et al., 2021).

**Fig. 1.**
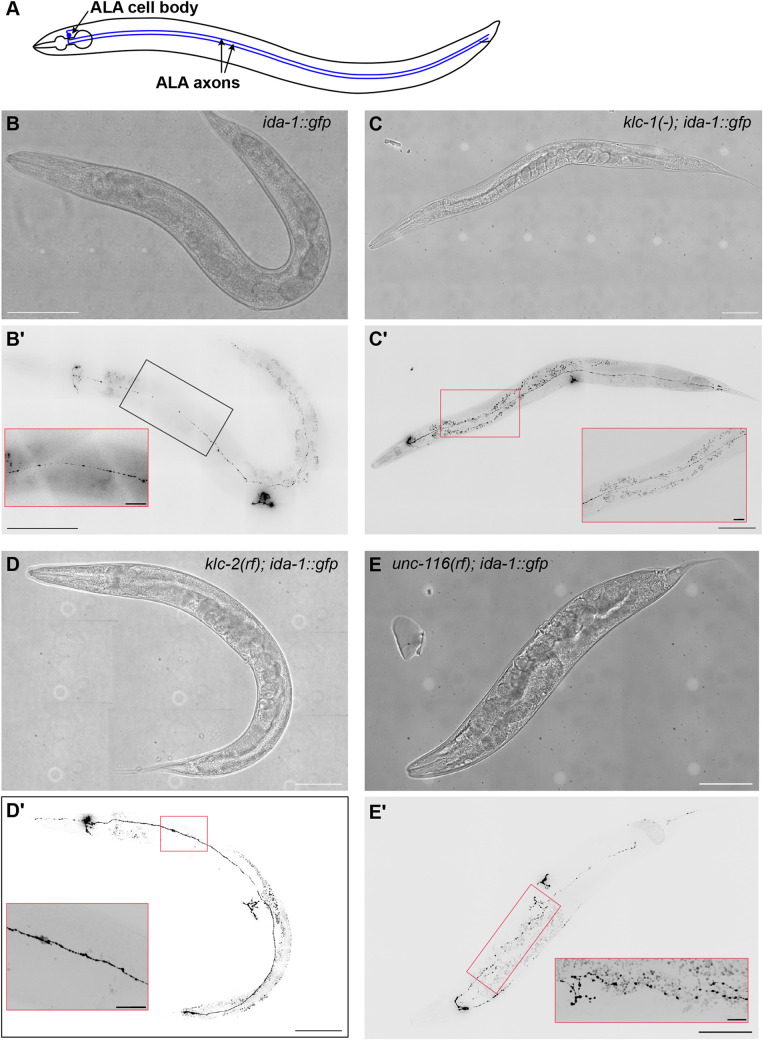
**Worm morphology and DCV distribution.** (A) Illustration of the ALA neuron. (B–E) Spinning disc brightfield and fluorescence images of *ida-1::gfp* (B), *klc-1(-); ida-1::gfp* (C), *klc-2(km11); ida-1::gfp* (D) and *unc-116(rf); ida-1::gfp* (E) worms showing the DCVs (inverted look-up table). Scale bars: 100 µm. An enlarged section of each ALA neuron is shown in the red boxes. The lighter spots in the brightfield images are artefacts generated by the tiling of multiple images. Scale bars: 20 µm. The larger particles visible outside the ALA neuron are auto-fluorescent granules, mainly located in the gut. Images were collected using a 20× objective for *ida-1::gfp* and a 40× objective for the other strains. Images representative of at least ten animals examined.

Here, we have investigated the function of kinesin-1 in DCV movement in *C. elegans*, using worms expressing GFP-tagged IDA-1 (‘islet cell diabetes autoantigen-1’) protein, which is the *C. elegans* orthologue of mammalian type-1 diabetes auto-antigen proteins insulinoma associated protein-2 (IA-2; also known as INSM2) and phogrin (IA-2^β^; also known as PTPRN2) (Cai et al., 2001; Zahn et al., 2001). Expression of IDA-1::GFP from its endogenous promoter has revealed that it is localised to DCVs in the ALA, VC, HSN and PHC neurons, and the uv1 neurosecretory cell (Cai et al., 2004; Zahn et al., 2004), although RNAseq and *ida-1*-promoter-driven GFP expression suggest *ida-1* is expressed more widely at lower levels (Cai et al., 2001; Cao et al., 2017; Taylor et al., 2021; Zahn et al., 2001). We imaged DCV motility in the ALA neuron, which has two axons that run the length of the worm in both lateral nerve tracts (Fig. 1A). It has relatively few chemical synapses, but instead both receives and transmits signals via neuropeptides (Iannacone et al., 2017; Konietzka et al., 2020; Nath et al., 2016; Nelson et al., 2014; Randi et al., 2023; Ripoll-Sanchez et al., 2023). Two peaks of anterograde DCV velocity have been reported in ALA neurons, with the faster being lost in an *unc-104* (kinesin-3) mutant (Zahn et al., 2004), again suggesting that another kinesin can drive DCV motility.

Kinesin-1 is a hetero-tetrameric molecular motor complex, consisting of two kinesin heavy chains (KHCs; KIF5 proteins in mammals) and two kinesin light chains (KLCs) (Cross and Dodding, 2019; Verhey and Hammond, 2009). In the genome of *C. elegans*, a single KHC is encoded by the *unc-116* gene, whereas two KLCs are encoded by the *klc-1* and *klc-2* genes (Sakamoto et al., 2005). KLCs play a crucial role in recruiting kinesin-1 to cargo and are actively involved in activating the motor (Anton et al., 2021; Chiba and Niwa, 2024; Chiba et al., 2022; Cross and Dodding, 2019). Most interactions between KLCs and cargo proteins occur via the KLC tetratricopeptide repeat (TPR) region, which is highly conserved between KLC isoforms and across species (Fig. S1; Anton et al., 2021; Gumusderelioglu et al., 2023). In *C. elegans*, KLC-1 is expressed at lower levels and in fewer cell types and tissues than KLC-2 (Taylor et al., 2021) and is less well studied. Whereas *unc-116* and *klc-2* null alleles are embryonic lethal (Byrd et al., 2001; Sakamoto et al., 2005), *klc-1* null animals are viable and appear superficially wild type (Consortium, 2012). KLC-1 and -2 appear to function redundantly in meiotic spindle positioning (Yang et al., 2005) and mitochondrial transport in some situations (Zhao et al., 2021) but not others (Ding et al., 2022; Sure et al., 2018), whereas only KLC-1 is needed for shedding of mitochondria in apoptotic oocytes (Raiders et al., 2018). In contrast, only KLC-2 is involved in transporting the glutamate receptor GLR-1 (Hoerndli et al., 2013). However, many studies have not compared the two KLCs.

To test the roles of the KLCs and KHC in DCV transport, we analysed the movement of IDA-1::GFP-labelled DCVs in three strains bearing mutations in the *klc-1*, *klc-2* and *unc-116* (the KHC) genes. We find that DCV movement in both directions is severely compromised by the *unc-116* mutation and partially affected by the *klc-2* mutation. Surprisingly, dynein-driven DCV motility is faster in the *klc-1* mutant than in the wild-type background, whereas plus-end-directed movement is unaffected. Because of the importance of neuropeptides in ageing and locomotion, we performed crawling, swimming (also called thrashing) and adult lifespan assays on these single and double mutants, and determined their sensitivity to aldicarb and levamisole, drugs that affect neuromuscular junction (NMJ) function. These revealed clear differences between the effects of *klc-1* and *klc-2* on locomotion and synaptic transmission at the NMJ. In addition, whereas the *klc-1* and *-2* mutant worms had the same lifespan as wild type, the *unc-116* mutant worms were short lived. The *ida-1::gfp* transgenic animals also had a shorter lifespan. Unexpectedly, expressing *ida-1::gfp* in either the *klc-1* or *klc-2* mutant strains gave animals with a considerably longer lifespan than wild-type worms or the parental strain. This highlights a novel role for kinesin-1 in the regulation of lifespan.

## RESULTS

To investigate kinesin-1 function in DCV motility, we crossed established kinesin-1 mutants with a strain expressing IDA-1::GFP from two integrated extrachromosomal arrays under the control of the *ida-1* promoter (giving ∼2–3-fold overexpression of IDA-1 protein) (Zahn et al., 2004). The strong hypomorphic kinesin heavy chain mutant *unc-116(rh24sb79)* (Patel et al., 1993; Yang et al., 2005) [referred to as *unc-116(rf)* herein, to indicate its reduced function] contains three missense mutations within the motor domain (Yang et al., 2005). The hypomorphic, reduced function kinesin light chain 2 mutant *klc-2(km11)* (Sakamoto et al., 2005) contains two copies of truncated *klc-2* genes. The endogenous *klc-2* locus carries a ∼1.4-kb deletion, which truncates the protein product immediately after the coiled-coil, eliminating the entire TPR region (Fig. S1). Additionally, a second copy of the *klc-2* gene, inserted upstream of the endogenous locus and in the opposite orientation, lacks the two alternative last exons, resulting in a protein that would be truncated after the TPR domains (Sakamoto et al., 2005), if it were to be expressed. We also used the kinesin light chain 1 null mutant *klc-1(ok2609)* (Consortium, 2012), referred to as *klc-1(-)*, which has the sequence QQTLFALRDEHEAATRILEANLISKTIYGMSTNKVSIRFRLIRTFFAQ-stop inserted after amino acid 36. Imaging IDA-1::GFP in these new strains revealed normal ALA morphology and DCV distribution in the *klc-1(-)* and *klc-2(km11)* backgrounds (Fig. 1B–D). In contrast, *unc-116(rf); ida-1::gfp* worms often displayed branching of the ALA axons (Fig. 1E; Fig. S2), with visual analysis identifying branching in 17 of 25 young adult worms.

### Effects of kinesin-1 mutants on DCV motility

**Fig. 2.**
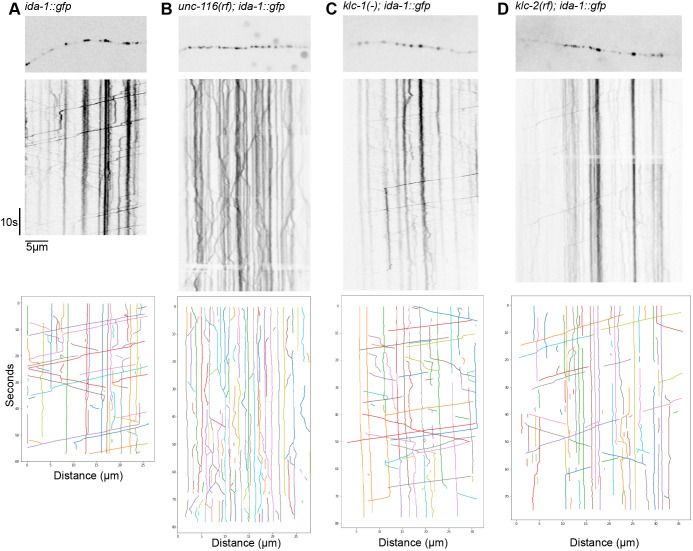
**Kymographs of DCV movement in the ALA neuron.** The initial frame of each movie is shown on top, with the kymograph below and the tracks identified by KymoButler at the bottom. (A) *ida-1::gfp*, (B) *unc-116(rf); ida-1::gfp*, (C) *klc-1(-); ida-1::gfp* and (D) *klc-2(km11); ida-1::gfp*. The nerve terminal (microtubule plus ends) is on the right. Kinesin-driven DCVs correspond to lines sloping from top left to bottom right, with dynein-driven lines sloping from top right to bottom left. Vertical lines indicate stationary DCVs (individual or clusters). Images representative of 10–17 animals and 53–127 kymographs examined (see Table 1 for full details).

We imaged DCVs in the ALA neuron by spinning disc confocal microscopy of single z-planes of worms immobilised by treatment with a mixture of tetramizole and tricaine (see Materials and Methods). From each image series, the position of DCVs over time was displayed as kymographs (Fig. 2; Fig. S2). Consistent with a previous report (Zahn et al., 2004), DCVs in *ida-1::gfp* worms moved in both directions, but rarely reversed (Fig. 2A; Fig. S3A, Movie 1). In addition, there were notable points where particle clusters did not move, which likely correspond to the periodic swellings observed by electron microscopy (EM) along the length of the ALA neuron containing DCVs, some of which might occur at *en passant* synapses (Ramirez-Suarez et al., 2019). Large IDA-1::GFP clusters have been shown to colocalise with synaptobrevin, a synaptic marker (Cai et al., 2004). When approaching such a cluster, the moving DCV could either stop or pass through. For anterograde DCVs, 75% moved through at least one cluster with 60% stopping at a cluster, either transiently or for a prolonged period (*n*=163 vesicles scored in 49 kymographs from eight experiments). Almost all (99.7%) retrogradely transported DCVs moved through clusters and 22.3% stopped at least once (*n*=332 DCVs scored). Clusters could also shed DCVs that then moved off in either direction, with 48% of released DCVs moving anterogradely and 19% moving retrogradely.

**Table 1.**
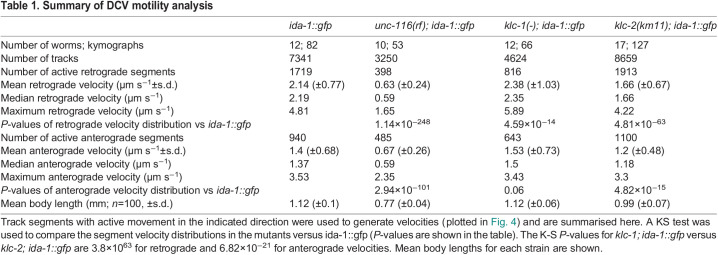
Summary of DCV motility analysis

**Fig. 3.**
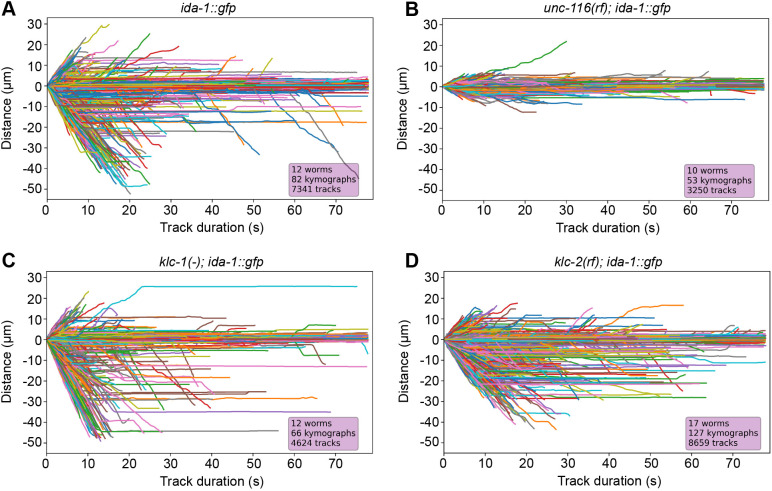
**Visualisation of individual DCV tracks in the ALA neuron.** Displacement plots (distance moved versus time) showing the movement of DCVs in the ALA neuron in (A) *ida-1::gfp*, (B) *unc-116(rf); ida-1::gfp*, (C) *klc-1(-); ida-1::gfp* and (D) *klc-2(km11); ida-1::gfp* worms. The number of worms, kymographs and tracks analysed are shown in the figure.

**Fig. 4.**
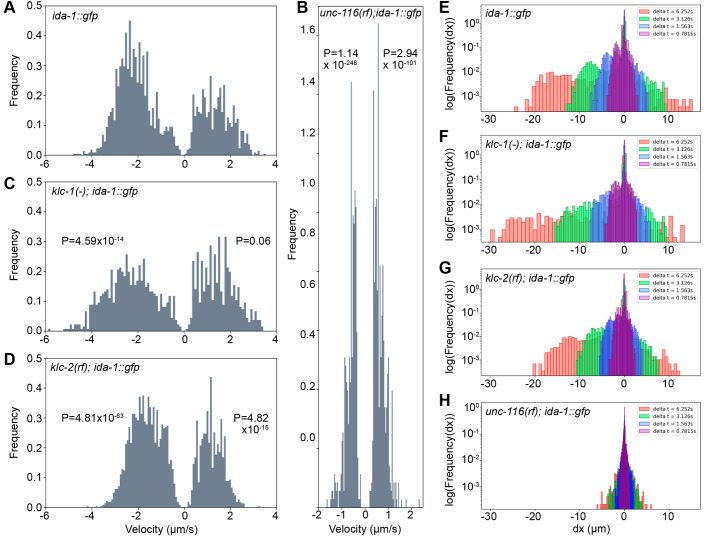
**Velocity and displacement distribution plots of DCV movement.** The distribution of velocities of active segments for DCV movement are plotted for (A) *ida-1::gfp*, (B) *unc-116(rf); ida-1::gfp*, (C) *klc-1(-); ida-1::gfp* and (D) *klc-2(km11); ida-1::gfp*. The probability density functions (frequency) were estimated from histograms with a bin size of 100; negative velocities are for dynein-driven movement; positive velocities are for movement towards the tail (microtubule plus ends). Note that the *y*-axis scale is the same in A–D. The number of segments used to generate velocities is given in Table 1*. P*-values from pairwise Kolmogorov–Smirnov tests with a Tukey–Kramer post-hoc honest significant difference test for velocity distribution of DCVs in the different strains are shown for both directions of movement (see also Table 1). This data is replotted as the distribution of displacements (on a log scale) within 5, 10, 20 or 40 frames (E–H).

To determine DCV velocities, kymographs were automatically tracked using KymoButler (Jakobs et al., 2019), then analysed using custom-written Python scripts. A plot of DCV displacement versus time for all tracks (Fig. 3A) revealed prominent dynein-driven motion with an average velocity of 2.14±0.77 µm s^−1^ whereas plus-end-directed movement had an average velocity of 1.4±0.68 µm s^−1^ (mean±s.d.) (Table 1). The distribution of velocities (Fig. 4A) broadly resembles that reported previously for a small number of DCVs in the ALA neuron (Zahn et al., 2004). Gaussian fitting of the distributions revealed a single peak for retrograde and three peaks for anterograde velocities (Fig. S4). Scoring the tracks according to their behaviour revealed that 21.5% of DCVs moved without pausing, 11.9% moved with pauses, and 66.6% of DCVs (or DCV clusters) were stationary for the duration of the movie (Fig. S5). In total, 12.2% of DCVs moved for some or all the time towards the plus ends, with 22.7% having dynein-driven runs.

Although DCVs were still distributed along the ALA axons in the *unc-116(rf)* mutant background, their motility was profoundly inhibited. Some movement still occurred in both directions, but it was hesitant (Figs 2B, 3B; Fig. S3B, Movie 2) and slower, with averages of 0.67±0.26 µm s^−1^ for plus-end-directed motion and 0.63±0.24 for dynein-driven motility (Table 1; Kolmogorov–Smirnov test versus *ida-1::gfp*, *P*=2.94×10^−101^ for anterograde, *P*=1.14×10^−248^ for retrograde) with a much narrower range of values (Fig. 4B; note that the *y*-axis scale is the same for graphs in A–D). Furthermore, the displacements were visibly shorter (Figs 2B, 3B) and there was an increase in the percentage of stationary DCVs with a corresponding decrease in both plus- and minus-end-directed DCV movement (Fig. S5B; Table 1). Gaussian fitting of the segment velocities showed very different distributions, with new, slow peaks in both directions (Fig. S4).

In contrast, DCV dynamics in the *klc-1(-)* and *klc-2(km11)* mutants were broadly similar to those of wild type (Fig. 2; Fig. S3, Movies 3 and 4). However, in *klc-2(km11)* the mean DCV velocity was reduced in both directions (1.2±0.48 µm s^−1^ plus-end-directed; 1.66±0.67 µm s^−1^ dynein-driven; Table 1), and the distribution of the individual segment velocities were significantly different to those in wild-type *ida-1::gfp* worms [Fig. 4D; Table 1; Kolmogorov–Smirnov (KS) test versus *ida-1::gfp*, *P*=4.82×10^−15^ for anterograde, *P*=4.81×10^−63^ for retrograde]. Gaussian fitting revealed the loss of the fastest plus-end-directed segments whereas the retrograde segment distribution now contained a slower peak (Fig. S4). The percentage of DCVs moving in each direction and those remaining stationary were, however, very similar (within ±1.5) to those in wild type (Fig. S5).

In contrast to the *klc-2(km11)* mutant, the complete loss of *klc-1* had only a small, statistically insignificant effect on plus-end-directed mean velocity and the distribution of segment velocities (Figs 3C, 4C; Table 1; KS test versus *ida-1::gfp*, *P*=0.06). Surprisingly, dynein-driven DCVs moved significantly faster in the *klc-1(-)* mutant than in wild-type worms, with an average velocity of 2.38±1.03 µm s^−1^ (Table 1). The velocity distribution plot (Fig. 4C) revealed an increase in the proportion of dynein-driven DCVs moving at very high speeds, and the maximum retrograde DCV velocity also increased from 4.81 to 5.89 µm s^−1^ (Table 1; KS test versus *ida-1::gfp*, *P*=4.59×10^−14^). The percentages of DCVs moving in each direction versus those that were stationary were only slightly different to those in wild-type and *klc-2(km11)* worms (Fig. S5C). As an alternative way of demonstrating the difference in behaviour of the fastest-moving DCVs, we replotted the data to show the number of DCVs that reached a given displacement after 5, 10, 20 or 40 frames (Fig. 4E–G). This clearly shows the greater distance travelled by dynein-driven DCVs within a set time in the *klc-1(-)* mutant compared to the other strains.

Given that kinesin-1 is a plus-end-directed motor that would transport DCVs from the cell body towards the axon tip, we wondered whether DCV motility changed with increasing distance from the cell body. We therefore separated the data into four groups according to where in the ALA axon it was collected, then plotted the displacement versus time for each track (Fig. S6). Given that the *unc-116(rf)* and *klc-2(km11)* worms have a shorter body length (Table 1), there were no kymographs collected more than 622 or 696 µm from the ALA cell body in *unc-116(rf)* and *klc-2(km11)* worms, respectively (Fig. S6B,D). This analysis showed that DCV behaviour did not appear to be affected by position along the axon.

### Effect of kinesin-1 mutations on *C. elegan*s swimming and crawling

**Fig. 5.**
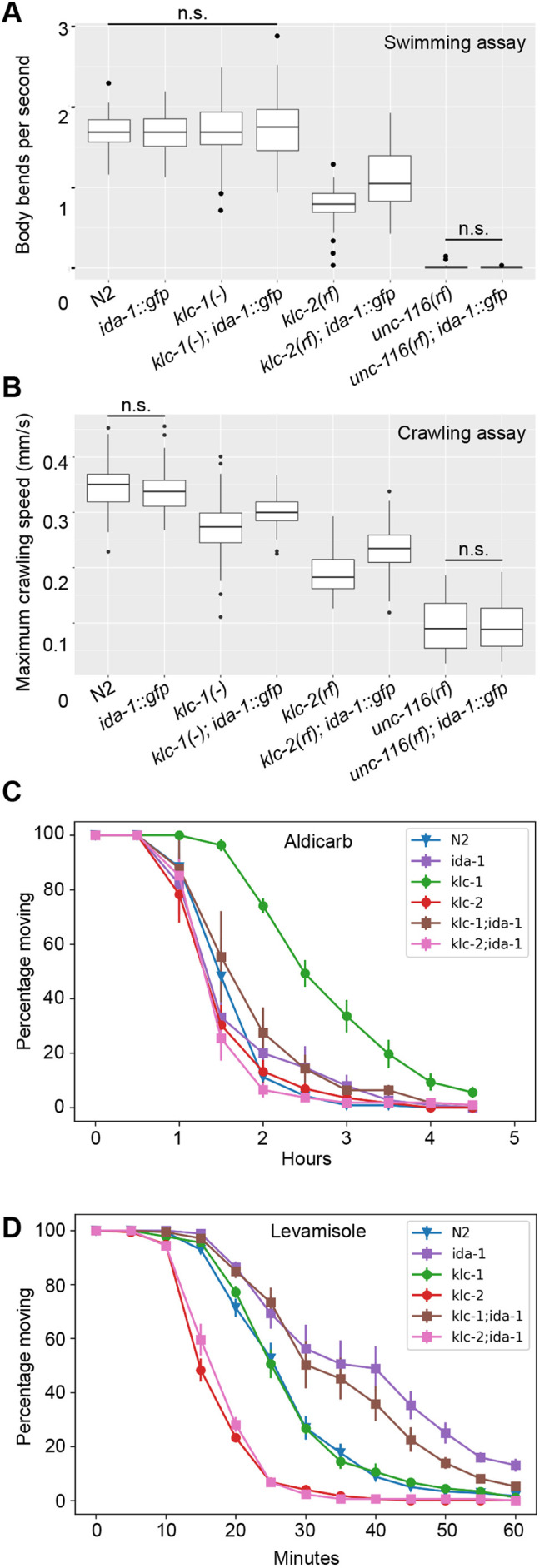
**Effect of kinesin-1 mutations and *ida-1::gfp* expression on the mobility of *C. elegans*.** (A) Body bends per second for N2 (*n*=49), *ida-1::gfp* (*n*=49), *klc-1(-)* (*n*=53), *klc-2(km11)* (*n*=30), *unc-116(rf)* (*n*=38), *klc-1(-); ida-1::gfp* (*n*=51), *klc-2(km11); ida-1::gfp* (*n*=49) and *unc-116(rf; ida-1::gfp)* (*n*=43) worms, analysed as day 1 adults in M9 buffer. (B) The maximum crawling speeds of the same strains were analysed for day 1 adults: N2 (*n*=29), *ida-1::gfp* (*n*=33), *klc-1(-)* (*n*=65), *klc-2(km11)* (*n*=71), *unc-116(rf) n*=55), *klc-1(-); ida-1::gfp* (*n*=49), *klc-2(km11; ida-1::gfp* (*n*=67), and *unc-116(rf); ida-1::gfp* (*n*=72). The boxplots display the median and two hinges, corresponding to the first and third quartiles. The whiskers extend from the hinge to the largest or smallest value within 1.5× the interquartile range (IQR) from the hinge. All ‘outlying’ points are plotted individually. Statistical analysis was performed by one-way ANOVA followed by a Tukey–Kramer post-hoc honest significant difference test, with results shown in Table S1 for swimming assays and Table S2 for crawling assays. All non-significant (n.s.) comparisons are indicated on the figure panels. (C,D) The time taken for day 1 adult worms to be paralysed by the acetylcholine esterase inhibitor aldicarb (C) or the acetylcholine agonist levamisole (D) was monitored (see Materials and Methods) for the following strains: N2, *ida-1::gfp*, *klc-1(-)*, *klc-2(km11)*, *klc-1(-); ida-1::gfp* and *klc-2(km11); ida-1::gfp*. The online application for survival analysis, OASIS 2 (Han et al., 2016), was used for analysis. A log-rank test with Bonferroni post-hoc correction was used to compare pairs of survival functions (Table S3). Experiments were repeated independently four times for aldicarb treatment and three times for levamisole treatment. The total number of worms analysed is given in Table S3. Plots show the mean±s.e.m. at each timepoint.

The *unc-116(rf)* mutant worms are very uncoordinated as well as having severely compromised DCV movement. Given that the *klc* mutants had distinct effects on DCV motility, we decided to test their effect on worm locomotion. We assessed swimming/thrashing ability by determining the number of body bends per second in M9 buffer (Fig. 5A; see Table S1 for statistical analysis) and measured the maximum speed of crawling on agar (Fig. 5B; see Table S2 for statistical analysis). Swimming and crawling are considered separate ‘gaits’ (Vidal-Gadea et al., 2011).

The swimming and crawling ability of *ida-1::gfp* worms was the same as wild-type worms. As expected, given their uncoordinated phenotype, the body bend frequency and crawling speeds of both *unc-116(rf)* and *unc-116(rf); ida-1::gfp* were extremely low and not significantly different from each other (Fig. 5; Tables S1, S2). The *klc-2(km11)* mutant on its own had a lower rate of body bends and crawling speed than N2 worms (*P*<10^−10^ for both), consistent with their slightly *unc* phenotype (Sakamoto et al., 2005). Surprisingly, however, both types of motion were improved significantly by the expression of the *ida-1::gfp* transgene in *klc-2(km11)* worms (*P*=8.59×10^−5^ for swimming; *P*=1.04×10^−6^ for crawling).

The *klc-1(-)* worm analysis showed that the swimming activity of both *klc-1(-)* and *klc-1(-); ida-1::gfp* worms was the same as wild type. However, the maximum crawling speed of both strains was significantly slower than N2 or *ida-1::gfp* worms, although faster than *klc-2* mutants (Fig. 5B; for statistical analysis, see Table S2). As was seen for *klc-2* mutants, expression of *ida-1::gfp* improved *klc-1(-)* crawling speeds (*P*=0.0236), although not back to wild-type levels (Fig. 5B; Table S2).

### Effect of *klc* mutations and *ida-1::gfp* expression on neuromuscular junction function

Given that *klc* mutants affected worm behaviour to varying extents, we wanted to determine whether they altered synaptic transmission at neuromuscular junctions (NMJs). Worm movement involves cholinergic neurons that promote muscle contraction on one side of the body while GABAergic neurons relax the muscles on the other side. The sensitivity of worms to paralysis by aldicarb, an acetylcholine (ACh) esterase inhibitor, and levamisole, an activator of one type of muscle acetylcholine receptors, are classic tests for NMJ function (Davis and Tanis, 2022; Mahoney et al., 2006). Aldicarb treatment causes ACh levels to build up in the synaptic cleft, over-stimulating the muscles and causing paralysis. Mutants with reduced ACh release are resistant to aldicarb because it takes longer for ACh to accumulate. For example, mutants in UNC-104, which delivers synaptic vesicles to the synapse (Hall and Hedgecock, 1991), are aldicarb resistant (Miller et al., 1996; Nguyen et al., 1995; Thapliyal et al., 2018). However, aldicarb resistance can also be due to increased GABA secretion (which relaxes muscles) or to decreased secretion of neuropeptides (Jacob and Kaplan, 2003; Miller et al., 1996; Mullen et al., 2006; Sieburth et al., 2005; Vashlishan et al., 2008). Levamisole resistance is caused by defects that reduce the response of the muscle to ACh, such as mutations in the levamisole-sensitive ACh receptor, UNC-63 (Lewis et al., 1980), whereas reduced GABA inhibitory signalling results in hypersensitivity (Vashlishan et al., 2008).

We assessed the sensitivity of all strains except *unc-116(rf)* and *unc-116(rf);ida-1::gfp*, which were not analysed because of their profoundly compromised mobility. Strikingly, *klc-1(-)* worms were aldicarb resistant (Fig. 5C; log-rank text *P*-values versus all other strains, <10^−10^, see Table S3 for full statistics) but responded to levamisole like N2 worms (Fig. 5D; Table S3). In contrast, the *klc-2(km11)* strain was hypersensitive to levamisole (*P*<10^−10^ versus N2) but had a normal response to aldicarb. These data suggest that the KLCs have distinct neuronal functions.

Expressing IDA-1::GFP in the wild-type background did not affect aldicarb sensitivity, in keeping with *ida-1::gfp* worm swimming or crawling ability being normal (Fig. 5). Even though the locomotion of *klc-2(km11); ida-1::gfp* worms was reduced compared to that of *ida-1::gfp* worms, the response of this strain to aldicarb was similar to that seen with *ida-1::gfp* alone (*P*=0.18). In contrast, the aldicarb resistance of *klc-1(-)* was greatly reduced by *ida-1::gfp* expression (Fig. 5C; *P*<10^−10^), although not quite back to N2 sensitivity (*P*=0.047; N2 versus *klc-1(-);ida-1::gfp*), as was seen for crawling ability (Fig. 5B; Table S2).

Surprisingly, however, expression of the *ida-1::gfp* transgene in the N2 background caused levamisole resistance (Fig. 5D; *P*<10^−10^ versus N2). This resistance was somewhat reduced in the *klc-1(-);ida-1::gfp* strain, although the difference was just short of statistical significance [Fig. 5D; *P*=0.0508, *ida-1::gfp* versus *klc-1(-);ida-1::gfp*]. The shape of both levamisole curves suggested that ∼40% of the population was particularly resistant to levamisole. In contrast, the *klc-2(km11);ida-1::gfp* strain was as hypersensitive to levamisole as *klc-2(km11)* (Fig. 5C,D; Table S3), indicating a dominant effect of the *klc-2* mutant.

Altogether, these results show that IDA-1::GFP expression is sufficient to modify post-synaptic NMJ function and influence the presynaptic and post-synaptic responses of *klc-1* mutants. In contrast, the *klc-2* mutation leads to levamisole sensitivity both in the single mutant and *ida-1::gfp*-expressing worms.

### Effect of kinesin-1 mutations and expression of the *ida-1::gfp* transgene on lifespan

**Fig. 6.**
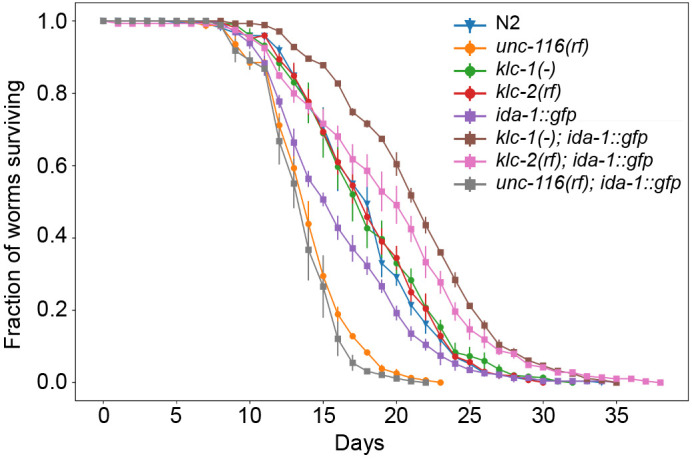
**Effect of kinesin-1 mutations and *ida-1::gfp* expression on the lifespan of *C. elegans*.** Survival plots showing the percentage of live worms as a function of time for N2, *ida-1::gfp*, *klc-1(-)*, *klc-2(km11)*, *unc-116(rf)* and the *klc-1(-)*, *klc-2(km11)* or *unc-116(rf)* strains expressing *ida-1::gfp*. Day 0 is the L4 stage of the worm. Each assay was repeated three times. Total number of worms scored were: N2=384, *ida-1::gfp*=390, *klc-1(-)*=390, *klc-2(km11)*=390, *unc-116(rf)*=387, *klc-1(-); ida-1::gfp*=384, *klc-2(km11); ida-1::gfp*=384 and *unc-116(rf); ida-1::gfp*=406. Error bars represent s.e.m. Results from log-rank tests with Bonferroni correction are shown in Table S4.

The genetic interaction between the *klc-2(km11)* and *klc-1(-)* mutations and the ida-1::gfp transgene led to enhanced worm motility in both backgrounds and improved NMJ function in the *klc-1(-)* background. This suggests improved health, which could translate into an increased lifespan (Hahm et al., 2015; Oswal et al., 2022). We therefore assessed lifespan in all kinesin-1 mutants alone and in the presence of *ida-1::gfp* (Fig. 6; see Table S4 for statistical analysis).

The wild-type N2, *klc-1(-)* and *klc-2(km11)* worms had similar lifespans (50% survival at day 18 for all strains) meaning that these kinesin light chain mutations do not affect longevity. Interestingly, the lifespan of *ida-1::gfp* worms (50% survival at 16 days) was shorter than N2 (*P*=2×10^−4^, log-rank test) suggesting that expression of this transgene has a negative impact on worm health as well as post-synaptic function, despite not affecting mobility or aldicarb sensitivity. Both *unc-116(rf)* and the *unc-116(rf); ida-1::gfp* strains had greatly reduced lifespans (50% survival at 14 days) compared to N2 and *ida-1::gfp* (*P*-values<10^−10^, log-rank test). Strikingly, however, expression of the *ida-1::gfp* transgene in either *klc* mutant gave a significant increase in lifespans compared to N2 or the *klc* single mutants (50% survival at 22 days for *klc-1; ida-1::gfp* and 20 days for *klc-2; ida-1::gfp*; *P*<10^−10^ for all comparisons with N2 and *klc* single mutants). Notably, these strains were dramatically longer lived than the *ida-1::gfp* parental strain (*P*<10^−10^), with the biggest effect seen in the *klc-1(-)* mutant background. Therefore, *klc-1(-)* and *klc-2(km11)* mutants genetically interact with the short-lived *ida-1::gfp* transgene strain, to increase lifespan beyond wild type.

## DISCUSSION

### Kinesin-1 is a motor for DCV movement

Axonal transport of large organelles, such as DCVs, through narrow calibre axons is a challenging process (Maday et al., 2014; Sabharwal and Koushika, 2019). It is well established that kinesin-3 motors – UNC-104 in worms and flies, and KIF1 in vertebrates – are key drivers of this movement, given that DCVs mainly get stuck in the neuron cell body when its function is disrupted (see Introduction). We show that kinesin-1 is also vital for DCV movement in the *C. elegans* ALA neuron, because DCVs move much more slowly and hesitantly in *unc-116(rf)* worms. Nevertheless, DCVs do enter the axon, are evenly distributed along its length, and still move in both directions to a degree. This contrasts with what is seen for two organelles that are transported solely by kinesin-1 – mitochondria and calsyntenin-positive vesicles – which remain in the neuronal cell body when kinesin-1 function is disrupted (Araki et al., 2007; Glomb et al., 2023; Konecna et al., 2006; Pilling et al., 2006; Rawson et al., 2014; Sure et al., 2018; Zhao et al., 2021). Altogether, these data fit with the hypothesis that both kinesin-1 and -3 are needed to drive DCV movement, with kinesin-3 being essential for exiting the cell body then both motors driving long distance movement (Arimura et al., 2009; Gumy et al., 2017; Lim et al., 2017; Park et al., 2023; Zahavi et al., 2021). Combining kinesins with different mechanochemical properties is thought to provide optimal cargo transport in complex cellular environments (Arpag et al., 2019; Gumy et al., 2017; Lim et al., 2017; Prevo et al., 2015).

### Effect of *unc-116* and *klc-2* mutants on DCV motility

The motility of DCVs in wild-type animals revealed a wide spread of plus-end-directed velocities that were fitted by three Gaussian distributions (Fig. S4). Similar distributions are seen for DCV movement in mouse and rat neurons (Gumy et al., 2017). Sophisticated analysis of Rab6-positive secretory vesicles in MRC5 cells has demonstrated that the slow movement is driven by kinesin-1 and the fast movement by kinesin-3, whereas the intermediate speed population has a mixture of the two motors (Serra-Marques et al., 2020). This fits with data showing that kinesin-1 is a slow motor whereas kinesin-3 motors are fast (Arpag et al., 2019, 2014; Glomb et al., 2023; Guedes-Dias et al., 2019; Gumy et al., 2017; Norris et al., 2014; Serra-Marques et al., 2020; Soppina et al., 2014), and implies that many DCV or secretory vesicle runs are driven by one motor or the other. The presence of a population with intermediate velocity could reflect both fast and slow motors engaged at once, or rapid switching between them, as previously suggested (Serra-Marques et al., 2020). Notably, however, the plus-end-directed velocities in the *unc-116(rf)* mutant, which still has wild-type UNC-104 motors, is drastically reduced to a velocity not seen in normal axons. Furthermore, in *klc-2(km11)* worms, the fastest population of velocities is lost, whereas the intermediate and slow distributions remain (Fig. S4). Although this might imply that kinesin-1 is faster than kinesin-3 *in vivo*, going against previous studies, the loss of the fastest DCV movement in ALA neurons in an *unc-104* mutant (Zahn et al., 2004) would suggest the opposite.

At the same time, the dynein-driven motion is also drastically slowed in *unc-116(rf)*, and a new slower peak of dynein velocities is seen in *klc-2(km11)*. This effect, termed ‘the paradox of co-dependence’ (Hancock, 2014) between kinesin and dynein, has been seen in many different situations, ranging from acute antibody-mediated inhibition of one motor (e.g. Brady et al., 1990; Uchida et al., 2009; Yi et al., 2011) through to function-modifying mutations, deletions or depletion (e.g. Ally et al., 2009; Encalada et al., 2011; Haghnia et al., 2007; Hoerndli et al., 2013; Iacobucci et al., 2014; Kwinter et al., 2009; Lim et al., 2017; Neisch et al., 2017; Vagnoni et al., 2016). The molecular basis for this relationship is still not understood.

Why, though, is UNC-104 not enough to continue to drive fast DCV movement when kinesin-1 (and dynein) function is reduced? The presence of the compromised UNC-116 motor (or partially inhibited dynein) might also act as a brake on UNC-104, effectively increasing the load and reducing the velocity it can achieve. In addition, given that kinesin-3 motors detach more readily from microtubules than kinesin-1 (Arpag et al., 2019, 2014; Norris et al., 2014), particularly under load, individual UNC-104 motors might be more likely to detach, therefore reducing the velocity an ensemble of motors can achieve. Indeed, this might explain why the DCV tracks in the *unc-116(rf)* mutant often appear to have velocities that fluctuate much more than the wild-type DCVs (Fig. 1A,B).

Although inhibition of kinesin-1 commonly reduces the speed of both directions (e.g. Hoerndli et al., 2013; Gumy et al., 2017; Ding et al., 2022), as we observe, normal dynein-driven rates have been seen in situations where kinesin-3 function is impaired (Hoerndli et al., 2013; Laurent et al., 2018; Ou et al., 2010; Zahn et al., 2004). This might fit with the observation that a kinesin-3 (KIF13B) on DCVs does not engage in a tug-of-war with dynein, whereas kinesin-1 does (Serra-Marques et al., 2020). However, other studies have reported reduced dynein DCV speeds in kinesin-3 mutants (Barkus et al., 2008; Edwards et al., 2015b; Lo et al., 2011). These differing results might be due to the different motor mutations or inhibition methods used and cargoes analysed. Approaches such as the rapid degradation of one motor type could provide new insight into how multiple motors work together on native cargoes.

### Loss of KLC-1 leads to faster dynein movement of DCVs

The null *klc-1* mutation did not affect the anterograde movement of DCVs. The *klc-1* gene is expressed at very low levels in the ALA neuron (Taylor et al., 2021) and its loss might be compensated for by KLC-2. Strikingly, however, the speed of retrograde transport actually increased in the absence of KLC-1. Faster dynein velocities have also been reported for UNC-104-driven synaptic vesicle precursor cargoes in the *unc-104(wy711)* mutant (Maeder et al., 2014) and a worm with a mutant neurofilament protein (Bhan et al., 2020), although in these cases the anterograde velocities were reduced.

How might KLC-1 loss enhance dynein-driven DCV motility specifically? Physical crowding within neurons is an important constraint on cargo motility (Maday et al., 2014; Sabharwal and Koushika, 2019), and the limited width of axons means that moving DCVs occupy most of that diameter (Ramirez-Suarez et al., 2019). One possibility is that absence of KLC-1 might change the physical environment in the ALA neuron in a way that facilitates dynein processivity indirectly. Interestingly, depolymerisation of actin increases DCV speeds in mouse cultured neurons, although directionality was not assessed (Bharat et al., 2017). It is also possible that KLC-1 in some way influences the balance of activity of dynein and kinesin-1, perhaps via a shared cargo adaptor, as has been recently reported for troponin-C regulation of *oskar* mRNA transport in *Drosophila* (Heber et al., 2024). For membrane transport, obvious candidate adaptors would be JIP proteins, which are known to bind both kinesin-1 and dynein, and control the transport of a range of different organelles into and out of axons (Arimoto et al., 2011; Celestino et al., 2022; Edwards et al., 2015a; Fu and Holzbaur, 2013; Cason and Holzbaur, 2022; Horiuchi et al., 2005; Sure et al., 2018). Whether worm KLC-1 influences any of these components or processes is an important question for future research.

### Effects of *klc* mutants and IDA-1::GFP expression on worm locomotion

Kinesin-1 is clearly required for worm locomotion, given the profoundly uncoordinated swimming and crawling of the *unc-116(rf)* mutant (Fig. 5). The *klc-2(km11)* reduced function mutant moderately affected both crawling and swimming whereas the complete loss of KLC-1 only had a mild effect on crawling. Although this might suggest redundancy between the KLCs, the responses of the *klc* mutants to the paralytic agents aldicarb and levamisole were completely distinct, arguing for at least some independent functions of kinesin-1 complexes containing KLC-1 compared to KLC-2.

The *klc-2(km11)* and *klc-2(km11); ida-1::gfp* mutants were both hypersensitive to levamisole, and the latter strain was also aldicarb sensitive (Fig. 5; Table S3). This behaviour is very similar to null mutants in glutamate receptor (GluR) subunits (Sadananda and Subramaniam, 2021). GluRs are expressed in the command interneurons that activate cholinergic motor neurons after receiving glutamate signals from mechanosensory neurons (Von Stetina et al., 2006). Importantly, the localisation and assembly of an AMPA-type GluR in the AVA command interneuron requires kinesin-1 and KLC-2 (Hoerndli et al., 2013, 2015), suggesting that reduced glutamatergic signalling explains the *klc-2(km11)* phenotypes we observe. Interestingly, AMPA-GluR function was normal in *klc-1(-)* animals (Hoerndli et al., 2013), but this might reflect the very low level of *klc-1* expression in the AVA neuron (Taylor et al., 2021) rather than an isoform-specific role for KLC-2 in AMPAR trafficking.

The *klc-1* gene is expressed most highly in the germ line (Taylor et al., 2021), in keeping with its role in meiotic spindle positioning (Yang et al., 2005) and mitochondrial transport out of apoptotic oocytes (Raiders et al., 2018). However, it is also expressed in a subset of neurons (Taylor et al., 2021) and we find that the *klc-1(-)* worms are resistant to aldicarb but not levamisole (Fig. 5), suggesting a presynaptic defect. Although aldicarb resistance is seen when ACh release is reduced, as in *unc-104* mutants (Miller et al., 1996; Nguyen et al., 1995; Thapliyal et al., 2018), this is unlikely to be the case in the *klc-1(-)* mutant, as *unc-116* mutants have normal synaptic vesicle precursor transport (Lipton et al., 2018). Alternatively, aldicarb resistance could be due to either increased GABA release or reduced neuropeptide secretion. Given that the GABAergic VD and DD motor neurons do not express *klc-1* (Taylor et al., 2021), it is more likely that neuropeptide signalling in cholinergic motor neurons is defective without KLC-1 due to impaired DCV delivery to synapses. Although this is not what we see in the ALA neuron studied here, cholinergic motor neurons express 10–23 times more *klc-1* than the ALA neuron (Taylor et al., 2021), contain motile DCVs (Goodwin and Juo, 2013; Goodwin et al., 2012), and express multiple neuropeptides and neuropeptide receptors (Ghaddar et al., 2023; Roux et al., 2023; Smith et al., 2024; Taylor et al., 2021). *klc-1* is also expressed in command interneurons and some mechanosensory neurons. Determining the role of KLC-1 in these neurons is an important topic for future research.

Our results clearly show that expression of the *ida-1::gfp* transgene (giving ∼2–3-fold more IDA-1 protein than normal; Zahn et al., 2004) improves the locomotion of both *klc* mutants and greatly diminishes the aldicarb sensitivity of *klc-1(-)* animals (Fig. 5). The *ida-1::gfp* strain is also levamisole resistant, with this resistance being slightly reduced in the *klc-1(-)* background, and transformed to levamisole sensitivity in the *klc-2(km11)* background. Previous work has implicated IDA-1 (and hence DCVs) in worm locomotion, given that the swimming ability of *ida-1* null worms is reduced (Cai et al., 2004; Fatima et al., 2014) and they are somewhat aldicarb resistant although they have wild-type sensitivity to levamisole (Cai et al., 2004). As the *ida-1* gene is expressed in 128 neurons to some level, including command interneurons, mechanosensory neurons and cholinergic motor neurons, although not GABAergic motor neurons (Taylor et al., 2021), a challenge for the future is to ascertain how IDA-1 and kinesin-1 work together to influence locomotion.

### Effects of kinesin-1 mutants and IDA-1::GFP expression on lifespan

There is also a clear genetic interaction between IDA-1 expression and kinesin-1 mutants in the context of lifespan. The expression of IDA-1::GFP shortened worm lifespan (Fig. 6), which likely relates to a role in insulin signalling. The insulin-IGF-1 signalling pathway controls lifespan in many species including *C. elegans* (Kenyon, 2010; Miller et al., 2020). The key components are the insulin-like peptides (ILPs) (Artan et al., 2016; Borbolis et al., 2020; Chen et al., 2013; Pandey et al., 2024), which bind to the DAF-2 insulin (IGF-1 in worms) receptor, downregulating the activity of the transcription factor DAF-16 and shortening life (Artan et al., 2016; Borbolis et al., 2020; Chen et al., 2013; Kenyon, 2010; Miller et al., 2020). Importantly, the ILPs are transported and secreted by DCVs. As higher IDA-1 expression levels correlate with an increased number of DCVs in the ventral cord region (Cai et al., 2009), the *ida-1::gfp* transgene strain might release more ILPs, leading to higher levels of IGF-1 signalling, so shortening lifespan.

Intriguingly, although the *klc* mutants had wild-type lifespan and the *ida-1::gfp* strain was short-lived, crosses between *ida-1::gfp* and the *klc* mutants extended lifespan. Given that anterograde DCV motility is reduced in the *klc-2(km11)* background, this could decrease delivery of DCVs and therefore secretion of IGF-1, so extending lifespan. In addition, UNC-116 and KLC-2 transport a splice form of the DAF2 insulin receptor, DAF2c, in neurons (Ohno et al., 2014). However, both these effects would also be expected in the *klc-2(km11)* single mutant as well, which has wild-type lifespan. Expressing the *ida-1::gfp* transgene in the *klc-1(-)* background gave the strongest extension of lifespan. Although KLC-1 is important for mitochondrial transport (Zhao et al., 2021), which could affect longevity in *klc-1(-); ida-1::gfp* worms through altered mitochondrial dynamics (Byrne et al., 2019; Morsci et al., 2016; Vagnoni et al., 2016; Zhang et al., 2019), again the single *klc-1(-)* mutant lifespan is normal. In contrast, *unc-116(rf)* worms died significantly sooner, and this was even worse when combined with *ida-1::gfp* expression. This could be due in part to impaired mitochondrial dynamics or DCV motility. There is also a correlation between worm mobility, healthspan and longevity (Hahm et al., 2015; Oswal et al., 2022). Indeed, the mild *unc-104(wy711)* allele shortened lifespan whereas expressing additional wild-type UNC-104 protein slightly extended lifespan in a pathway downstream of the DAF-2 IGF-1 receptor (Li et al., 2016).

The neurons responsible for the interplay between kinesin-1 function and *ida-1* expression levels in determining lifespan remain to be identified. However, *klc-1* expression is high in the NSM neuron (Taylor et al., 2021), which controls lifespan in the context of cold stress (Zhang et al., 2018), and in the ASJ neuron, which responds to dietary restriction (Artan et al., 2016). These neurons also express *ida-1* (Taylor et al., 2021). The ALA neuron is also interesting in the context of lifespan, as it controls a form of sleep that is induced by secretion of EGF and multiple neuropeptides and which occurs during larval development and following periods of stress (Iannacone et al., 2017; Konietzka et al., 2020; Nath et al., 2016; Nelson et al., 2014; Van Buskirk and Sternberg, 2007).

Taken together, these results show that kinesin-1 function is important for normal lifespan, and that it interacts in complex ways with the overexpression of a DCV component that is crucial for neuropeptide secretion. Where, and how, this interplay occurs remains to be determined, but it emphasises the importance of DCV function and motility, which we demonstrate relies on kinesin-1, as well as kinesin-3, in *C. elegans*, as it does in other species.

## MATERIALS AND METHODS

### *C. elegans* culture, maintenance and genetics

All *C. elegans* strains were maintained at standard conditions as previously described (Brenner, 1973); worms were kept at 20°C on 6 cm plates filled with nematode growth medium agar [NGM; 50 mM NaCl, 0.25% (w/v) Bacto-Peptone, 1.7% (w/v) agar, 1 mM CaCl_2_, 1 mM MgSO_4_, 25 mM KH_2_PO_4_, 5 µg/ml cholesterol] plates with OP-50 bacteria [*Caenorhabditis* Genetics Centre (CGC)] as food.

The *klc-1(ok2609)* (The *C. elegans* Deletion Mutant Consortium, 2012) and *klc-2(km11)* (Sakamoto et al., 2005) strains were obtained from the CGC. The *ida-1::gfp* originated from John Hutton's laboratory, University of Colorado Health Sciences Center, Denver, USA, and was kindly provided by Howard Davidson. The strain *unc-116(rh24sb79)* (Yang et al., 2005) was a gift from Frank McNally at the University of California-Davis, USA. Crosses were generated for this study in the Poulin laboratory. The N2 Bristol variety was used as a wild-type reference strain. All strains used in this study are listed in Table S5. Strains generated in this study will be made available via the CGC.

### Worm genotyping by single-worm PCR

To verify worm mutations and check successful crosses we used single worm PCR with different primers for each mutation. The primers used to identify the *klc-1(ok2609)* mutation are taken from the CGC: the outer left sequence is 5′-AAATGGCGCCAAGAGATATG-3′, the outer right sequence is 5′-TCACGCAATTTTGAGTTCGT-3′. To follow the *klc-2(km11)* deletion, we used KLC-2-f4 (5′-CGCCACGATCTCCTGTATTTCAATAGC-3′) and KLC-2-r4 (5′-GATGACGGAGTACAATGTCGAGCAAC-3′) primers (Sakamoto et al., 2005). To identify the *unc-116* mutation in crosses, we used its strong uncoordinated phenotype.

### Determination of lifespan

To perform lifespan experiments, 100–130 L4 worms for each strain were divided between six NGM OP50 plates and were monitored daily. To verify the result, we performed three rounds of the experiment (3150 worms in total). The worms were moved to new plates if they had progeny or if the plate became contaminated. Each day the number of live, dead and censored worms was scored. Reasons for censoring were: the worm died on the wall of the dish; the worm crawled into the agar; a bag of worms (progeny did not hatch and develop inside the mother); exploding/bursting vulva. Data were analysed using the OASIS 2 online application for survival analysis, which also performs statistical tests on the data (Han et al., 2016).

### Imaging and data analysis

Time-lapse microscopy of worms expressing IDA-1::GFP was used to capture DCV transport events. Worms were mounted in a paralyzing solution of 0.02% tetramisole hydrochloride (T1512, Sigma-Aldrich) and 0.2% tricaine MS-222 (3-amynobenzoic acid ethyl ester, A5040, Sigma-Aldrich) for at least 40 min before imaging to ensure proper immobilisation. Up to ten worms were placed in a drop of paralyzing solution on a 2% agarose pad on a microscope slide, then covered with an 18 mm coverslip. The edges of the coverslip were sealed with VALAP (a 1:1:1 mixture of vaseline, lanolin and paraffin) to prevent evaporation.

Confocal imaging was performed on a 3i inverted Spinning Disk Confocal Microscope and a motorised stage. Images were acquired using a CSU-X1 spinning disc confocal (Yokogawa) on a Zeiss Axio-Observer Z1 microscope with a 20×/0.5 EC PlanNeofluar, 40×/1.30 Plan-Apochromat (Oil; DIC), 63×/1.40 Plan-Apochromat and 100×/1.30 Plan-Neofluar objectives, Prime 95B Scientific CMOS (1200 x1200 11 µm pixels; backlit; 16-bit) camera (Photometrics) and motorised *xyz* stage (ASI). The 488 nm laser was controlled using an AOTF through the laser stack (3i) allowing both rapid ‘shuttering’ of the laser and attenuation of the laser power. SlideBook software (3i) was used to capture images.

To capture images of whole worms, ‘Multiple XY Location Capture in SlideBook’ was used to generate montage images (Fig. 1; Fig. S2). Montage fluorescence images were generated automatically, and montage brightfield images were generated manually. Brightfield and fluorescence images were taken with the same camera at 100 ms exposure, with LED brightfield illumination. Brightfield images are in one focal plane, whereas fluorescence images have several *z*-planes combined by maximum projection.

Videos of DCV movement collected using the 100× objective were made using a single plane with 156 ms frame interval for 500 frames. Raw TIFF files were stabilised using image stabilizer plugin for ImageJ (http://www.cs.cmu.edu/~kangli/code/Image_Stabilizer.html) using the best suitable initial reference slice. For sections where stabilisation did not work, for example if the worm moved significantly, the frames were deleted. Standard parameters for image stabilizer were used. A segmented line was then superimposed over the neuron in the stabilised videos and the ImageJ Multi Kymograph plugin (https://biii.eu/multi-kymograph) was used to create kymographs. The best quality kymographs were chosen for subsequent analysis: 82 of *ida-1::gfp*, 66 kymographs of *klc-1(-); ida-1::gfp*, 127 kymographs of *klc-2(km11); ida-1::gfp*, and 53 kymographs of *unc-116(rf); ida-1::gfp*. Tracks were identified using KymoButler (Jakobs et al., 2019), with examples shown in Fig. 2 and Fig. S3. The KymoButler code was obtained from https://github.com/MaxJakobs/KymoButler/ and run in Mathematica software (Wolfram) to give the *x* and *t* positions for each track in every kymograph.

For each strain, we combined KymoButler results to analyse and visualise results using custom-written Python code (available at https://github.com/umkich/organelle_transport_analysis). In cases where two *x* positions corresponded to the same *t*, indicating that a particle occupied two positions simultaneously, which is impossible, the average position was computed. As KymoButler results are in pixels, they were next converted into seconds and µm. In Fig. 3, all tracks for each strain were combined with one starting point to visualise transport properties and compare them across different strains.

To compare retrograde and anterograde movement, each track was divided into segments: retrograde, anterograde and stationary, using a sliding window algorithm with a 0.6444 µm movement threshold and a 2 s time threshold. Each track can have multiple segments of each type (see Fig. S5E for examples). Venn diagrams were employed to illustrate the distribution of tracks exhibiting retrograde, anterograde, stationary or combinations of segment types (Fig. S5). Velocities were computed for retrograde or anterograde segments by dividing the distance displacement by the time displacement. Segments with fewer than four points were omitted from velocity calculations to enhance robustness [*ida1::gfp*, 37 tracks (0.5%); *klc-1(-); ida-1::gfp*, 21 tracks (0.45%) and *klc-2(km11); ida-1::gfp*, 28 tracks (0.32%)]. No tracks were excluded for *unc-116(rf); ida-1::gfp*. The velocities of retrograde or anterograde segments were averaged to derive the mean and s.d., along with the median velocity and maximum retrograde or anterograde velocities. The velocity distribution patterns were analysed using Gaussian Mixture Models (GMMs). The analysis was conducted separately for retrograde and anterograde movements using the Python scripts described above (https://github.com/umkich/organelle_transport_analysis). For each strain and direction, the Akaike Information Criterion (AIC) was used to determine the optimal number of components for the GMM.

### Behavioural and drug sensitivity assays

Swimming (also called thrashing) and crawling assays were performed using a Leica M165 FC Fluorescence Stereo Microscope, an optiMOS™ Scientific CMOS Camera and a PLANAPO 1.0× objective. For crawling assays, worms were transferred to fresh NGM 3.5 cm plates without OP50. For swimming assays, worms were imaged in 1 ml of M9 buffer (42 mM Na_2_HPO_4_, 22 mM KH_2_PO_4_, pH 7.0, 85.5 mM NaCl, 1 mM MgSO_4_) added to NGM plates. Image streams consisting of 1000 frames with 15–17 ms exposure were captured using Micro-Manager (https://micro-manager.org/). The videos were analysed using the Stack Deflicker (https://www.phage.dk/plugins/deflicker.html) and wrMTrck plugins (https://www.phage.dk/plugins/wrmtrck.html) in Fiji software to quantify body bends per second or crawling speed for each worm. Subsequently, the data from multiple videos was combined for each strain.

Aldicarb assays were performed according to a detailed published protocol (Mahoney et al., 2006). Briefly, 3.5 cm NGM plates containing 1 mM aldicarb (Merck, 33386) were prepared and stored at 4°C. Four experimental repeats were performed using the same batch of plates, with the same investigator, who was unaware of the experimental conditions, scoring the assays. Plates were warmed to room temperature and a patch of OP50 bacteria was spotted in the centre and allowed to dry. A total of 30 day-1 adults of each strain were placed on each plate at 5-min intervals and scored for paralysis every 30 min. Worms were deemed to be paralysed if they did not respond to three touches on the head and the tail with a worm pick (Mahoney et al., 2006).

Effects of levamisole on swimming was carried using a modification of a detailed protocol (Davis and Tanis, 2022). 12-well plates were prepared with 2 ml of NGM per well, then seeded with OP-50 bacteria. Several days later, ∼15 L4 worms were added to each well, with 4 wells per strain (58–63 worms per strain in each experiment). The next day, 2 ml of 0.4 mM levamisole (Sigma, 31742) in M9 buffer was added to each well of one plate at 20 s intervals. Motility of the worms was then assessed visually every 5 min for 60 min. Once that assay was complete, the second 12-well tray was assayed. The experimenter did not know which strain was in each well. The experiment was repeated three times on different days. Data from aldicarb and levamisole assays was analysed using OASIS 2 (Han et al., 2016).

To analyse the morphology of the ALA neuron in strains expressing IDA-1::GFP, worms were transferred to a drop of 10 mM tetramisole (MP Biomedicals, 02152119) in M9 buffer on a 2% agarose pad, covered with a 18×18 mm #1.5 coverslip, which was then held in place with VALAP. ALA axon branching was scored visually in 25–30 worms of each strain on an Olympus IX-71 microscope with a 100×/1.35 NA UPlanApo objective. A 470 nm LED (Cairn Research) and a GFP filter cube was used to Illuminate samples, which were observed using a Prime 95B sCMOS camera (Photometrics) and MetaMorph (Molecular Devices) software.

### Statistical analysis

Velocity distributions of DCVs in the different strains were compared by the pairwise two-sample Kolmogorov–Smirnov (KS) test (Table 1). The null hypothesis for the KS test (the two distributions come from the same population) was rejected at the *P*=0.05 level. To test the significant differences in the body bends per second or maximum crawling velocity for each pair of strains in the swimming and crawling assays (Fig. 5), one-way ANOVA was performed followed by Tukey–Kramer post-hoc tests (Tables S1 and S2). Sensitivity to aldicarb and levamisole (Fig. 5C,D), and lifespan survival functions (Fig. 6), were compared by the log-rank test with Bonferroni correction (Tables S3, S4) with the null hypothesis of no difference in survival between two groups at the significance level of 0.05, using OASIS 2 (Han et al., 2016).

## Supplementary Material



10.1242/joces.262148_sup1 Supplementary information
