# Supplementary material for: The role of kinesin-1 in neuronal dense core vesicle transport, locomotion and lifespan regulation in C. elegans
Source: J Cell Sci. 2024 Sep 6;137(17):jcs262148. doi: 10.1242/jcs.262148 (PMC11423817; doi:10.1242/jcs.262148)
Supplement: Supplementary information [file joces-137-262148-s1.pdf]

A

```
ceKLC-1   MVISLGDDITTVLKTVQQTLFALRDEHEAATRILEANLINSDSSEPSLPSEKMGLIDESLGKVMDGGDEASLLIMMDKLM 80
ceKLC-2   MSNMSQDDVTTGLRTVQQGLEALREEHSTISNTLETSVKGVKEDEAPLPKQKLSQINDNLDKLVCGVDETSLMLMVFQLT 80
          *     **:** *:**** * ***:**.: :. **:.: . ...*  **.:*:. *::.*.*:: * **:**::*: :*

ceKLC-1   QSYDVQLSKNHESIRLLRQENTWLLDELTTTQRKLQESERTVAHLEEERDHYKFQDSMNYLNSDF----QHTTSVDATPM 156
ceKLC-2   QGMDAQHQKYQAQRRRLCQENAWLRDELSSTQIKLQQSEQMVAQLEEENKHLKYMASIKQFDDGTQSDTKTSVDVGPQPV 160
          *. *.* .* : . * * ***:** ***::** ***:**: **:****..* *:  *:: ::..     : :..*.  *:

ceKLC-1   MVDTLQELGFGPEEEDQNN-NQADQGCRSSSFSNPISNDYQLPTRLQTLQNLVIQYMEQGRFEVAIPLCKQALEDVVKVH 235
ceKLC-2   TNETLQELGFGPEDEEDMNASQFNQPTPANQMAASANVGYEIPARLRTLHNLVIQYASQGRYEVAVPLCKQALEDLEKTS 240
            :**********:*:: * .* :*   :..::    . .*::*:**:**:****** .***:***:*********: *.

ceKLC-1   GNVHLDVATMLNVLAIVYRNQENFKDAAIYLEKALSIRVQCCGENHHSVAATLNNLAIAYGKRGKYKESEPLCKRALEIR 235
ceKLC-2   GHDHPDVATMLNILALVYRDQNKYKEAANLLNEALSIREKCLGESHPAVAATLNNLAVLFGKRGKFKDAEPLCKRALEIR 240
          *: * *******:**:***:*:::*:**  *::***** :* **.* :*********: :*****:*::***********

ceKLC-1   KNLLGPNHPDVAKQLTNLGIVTQQLEKYEETENYFKQALSIYNRAFPENHQNVIKTKNQLASVFLKQGKYQEAEEMYKNI 355
ceKLC-2   EKVLGDDHPDVAKQLNNLALLCQNQGKYEEVEKYYKRALEIYESKLGPDDPNVAKTKNNLSSAYLKQGKYKEAEELYKQI 360
          ::** :********.**.:: *:  ****.*:*:*:**.**:   :. ** ****:*:*.:******:****:**:*

ceKLC-1   LSKVA-------ITGNKPIWRIAEDREERQRNGIPKVDDESFNVNP-TTVMDSNVMSTIKNLAAVYRKQGKEEAAGTLEE 467
ceKLC-2   LTRAHEREFGQISGENKPIWQIAEEREENKHKGEGATANEQAGWAKAAKVDSPTVTTTLKNLGALYRRQGKYEAAETLED 480
          *::.          *****:***:***.::*   . :*. .    :.* . .* :*:***.*:**:*** *** ***:

ceKLC-1   ALGA-KKQI-------NGGADHTNST--ASSV-ETSSAVAINPAQSGIKKRIMHFVGLNF                    516
ceKLC-2   VALRAKKQHEPLRSGAMGGIDEMSQSMMASTIGGSRNSMTTSTSQTGLKNKLMNALGFNS                    540
          .    ***          ** *. ..:  **::  : .::: . :*:*:*:::*: :*:*
```

B

```
                  ---------------TPR1---------------      ---------------TPR2------------------
ceKLC-1   DYQLPTRLQTLQNLVIQYMEQGRFEVAIPLCKQALEDVVKVHGNVHLDVATMLNVLAIVYRNQENFKDAAIYLEKALSIR
ceKLC-2   GYEIPARLRTLHNLVIQYASQGRYEVAVPLCKQALEDLEKTSGHDHPDVATMLNILALVYRDQNKYKEAANLLNEALSIR
hsKLC1    GYEIPARLRTLHNLVIQYASQGRYEVAVPLCKQALEDLEKTSGHDHPDVATMLNILALVYRDQNKYKDAANLLNDALAIR
hsKLC2    GYEIPARLRTLHNLVIQYASQGRYEVAVPLCKQALEDLEKTSGHDHPDVATMLNILALVYRDQNKYKEAAHLLNDALAIR
          .*::*:**:**:****** .***:***:*********: *. *: * *******:**:***:*:::*:**  *:.**:**

          --      --------------TPR3--------------    ----------------TPR4------------------
ceKLC-1   VQCCGENHHSVAATLNNLAIAYGKRGKYKESEPLCKRALEIRKNLLGPNHPDVAKQLTNLGIVTQQLEKYEETENYFKQA
ceKLC-2   EKCLGESHPAVAATLNNLAVLFGKRGKFKDAEPLCKRALEIREKVLGDDHPDVAKQLNNLALLCQNQGKYEEVEKYYKRA
hsKLC1    EKTLGKDHPAVAATLNNLAVLYGKRGKYKEAEPLCKRALEIREKVLGKDHPDVAKQLNNLALLCQNQGKYEEVEYYYQRA
hsKLC2    EKTLGKDHPAVAATLNNLAVLYGKRGKYKEAEPLCKRALEIREKVLGKFHPDVAKQLSNLALLCQNQGKAEEVEYYYRRA
           :  *:.* :*********: :*****:*::***********::**  ********.**.:: *:  * **.* *:::*

          -------    -------------------TPR5--------------------
ceKLC-1   LSIYNRAFPENHQNVIKTKNQLASVFLKQGKYQEAEEMYKNILSKVA-------ITGNKPIWRIAEDREERQRNGIPKV-
ceKLC-2   LEIYESKLGPDDPNVAKTKNNLSSAYLKQGKYKEAEELYKQILTRAHEREFGQISGENKPIWQIAEEREENKHKG-EGAT
hsKLC1    LEIYQTKLGPDDPNVAKTKNNLASCYLKQGKFKQAETLYKEILTRAHEREFGSVDDENKPIWMHAEEREECKGKQKDGTS
hsKLC2    LEIYATRLGPDDPNVAKTKNNLASCYLKQGKYQDAETLYKEILTRAHEKEFGSVNGDNKPIWMHAEEREESKDKRRDSAP
          *.**   :  :. ** ****:*:* :*****::** :**:**:.            *****  **:*** : :    .

                          ---------------TPR6----------------
ceKLC-1   --DDESFNVNPTTVMDSNVMSTIKNLAAVYRKQGKEEAAGTLEEALGAKK--
ceKLC-2   ANEQAGW-AKAAKVDSPTVTTTLKNLGALYRRQGKYEAAETLEDVALRAKKQ
hsKLC1    FGEYGGW-YKACKVDSPTVTTTLKNLGALYRRQGKFEAAETLEEAAMRSRKQ
hsKLC2    YGEYGSW-YKACKVDSPTVNTTLRSLGALYRRQGKLEAAHTLEDCASRNRKQ
            :  .:  :  .* . .* :*::.*.*:**:*** *** ***:      :
```

Fig. S1. KLC sequence alignments. A) *C. elegans* KLC-1 and KLC-2 sequence alignments. Highlighted text shows the heptad repeats (green), the LFP regulatory motif (yellow) and the TPR domains (blue). The amino acids of each TPR are shown in bold text. B) A comparison of *C. elegans* KLC-1 and KLC-2 with *H. sapiens* KLC-1 and -2. The TPRs are indicated in bold text and lines above. The red amino acids are those shown to be involved in the interaction between hsKLC2 and SKIP (SifA-kinesin interacting protein) (Pernigo et al., Science 340:356, 2013). The green highlights show amino acids required for Y-acidic motif interactions, which are found only in hsKLC1, not hsKLC2 (Pernigo et al., eLife 7:e38362, 2018). *C. elegans* KLC-2 would be able to interact with Y-acidic cargo, but it is not clear if that is true for KLC-1. Amino acid identity (*), high similarity (:) and weak similarity (.) are indicated below the sequence.

Journal of Cell Science • Supplementary information

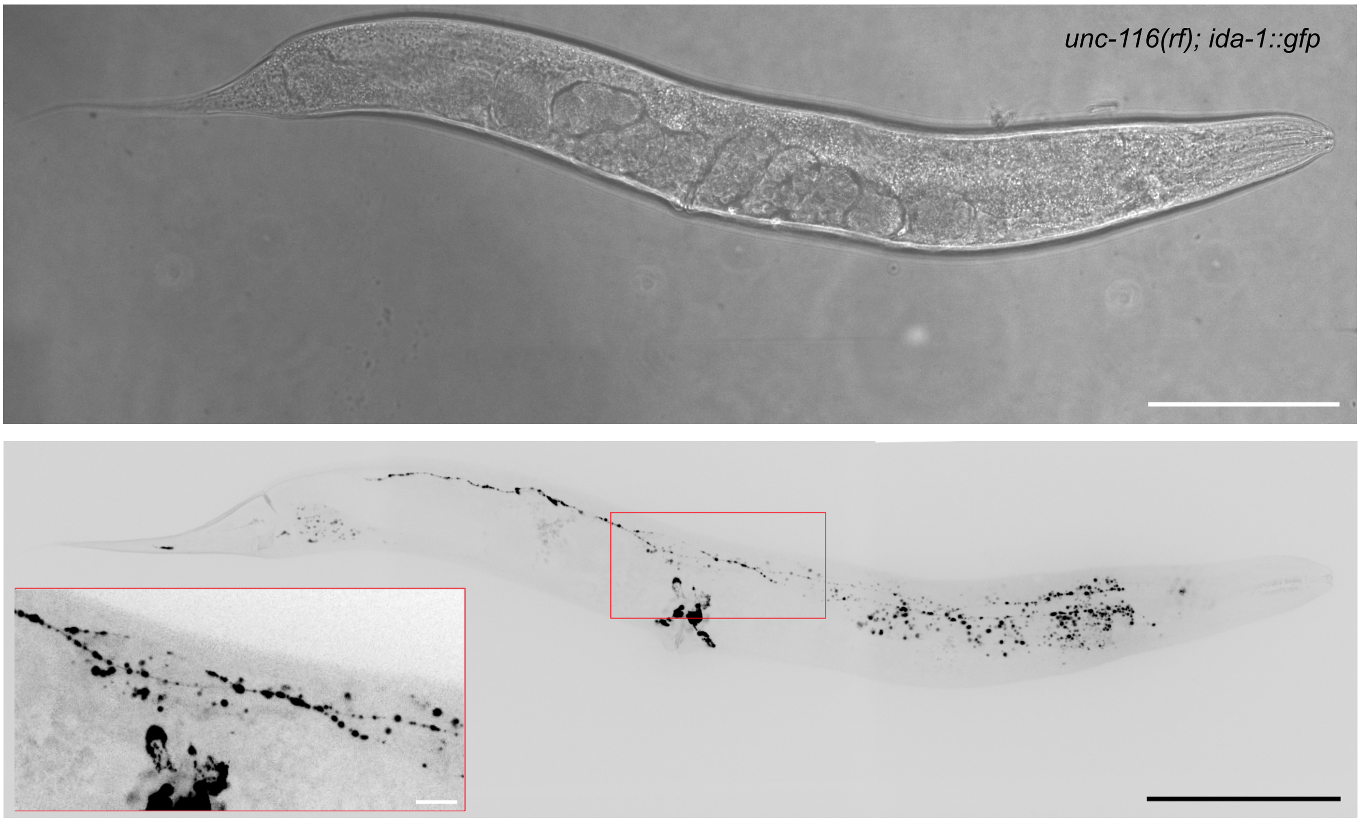

Fig. S2. Additional brightfield and fluorescence images of the *unc-116(rf); ida-1::gfp* strain. An enlarged section of each ALA neuron is shown in the red box. The lighter spots in the brightfield image are artefacts generated by the tiling of multiple images. The larger particles visible outside the ALA neuron are auto-fluorescent granules, mainly located in the gut. Scale bars are 100 µm with a 10 µm scale bar in the inset.

Journal of Cell Science • Supplementary information

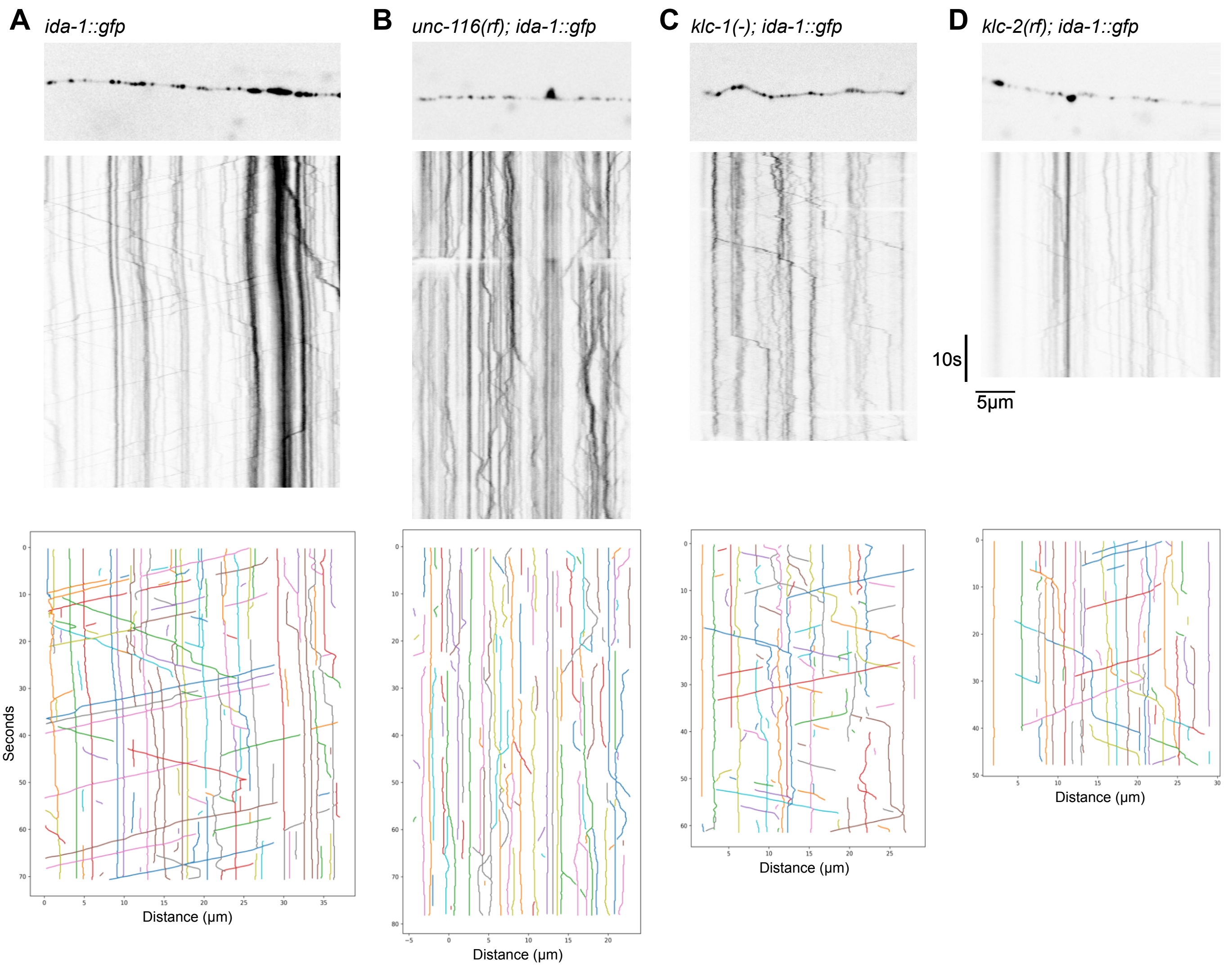

Fig. S3. Additional kymographs of DCV movement in the ALA neuron in different strains. The initial frame of each movie is shown on top, with the kymograph below. A) *ida-1::gfp*, B) *klc-1(-); ida-1::gfp*, C) *klc-2(rf); ida-1::gfp*, and D) *unc-116(rf); ida-1::gfp*. The nerve terminal (microtubule plus ends) is on the right for each kymograph. Kinesin-driven motile DCVs correspond to lines sloping from top left to bottom right, with dynein-driven lines sloping from top right to bottom left. Vertical lines indicate stationary DCVs (individual or clusters). Scale bars are 5 μm.

Journal of Cell Science • Supplementary information

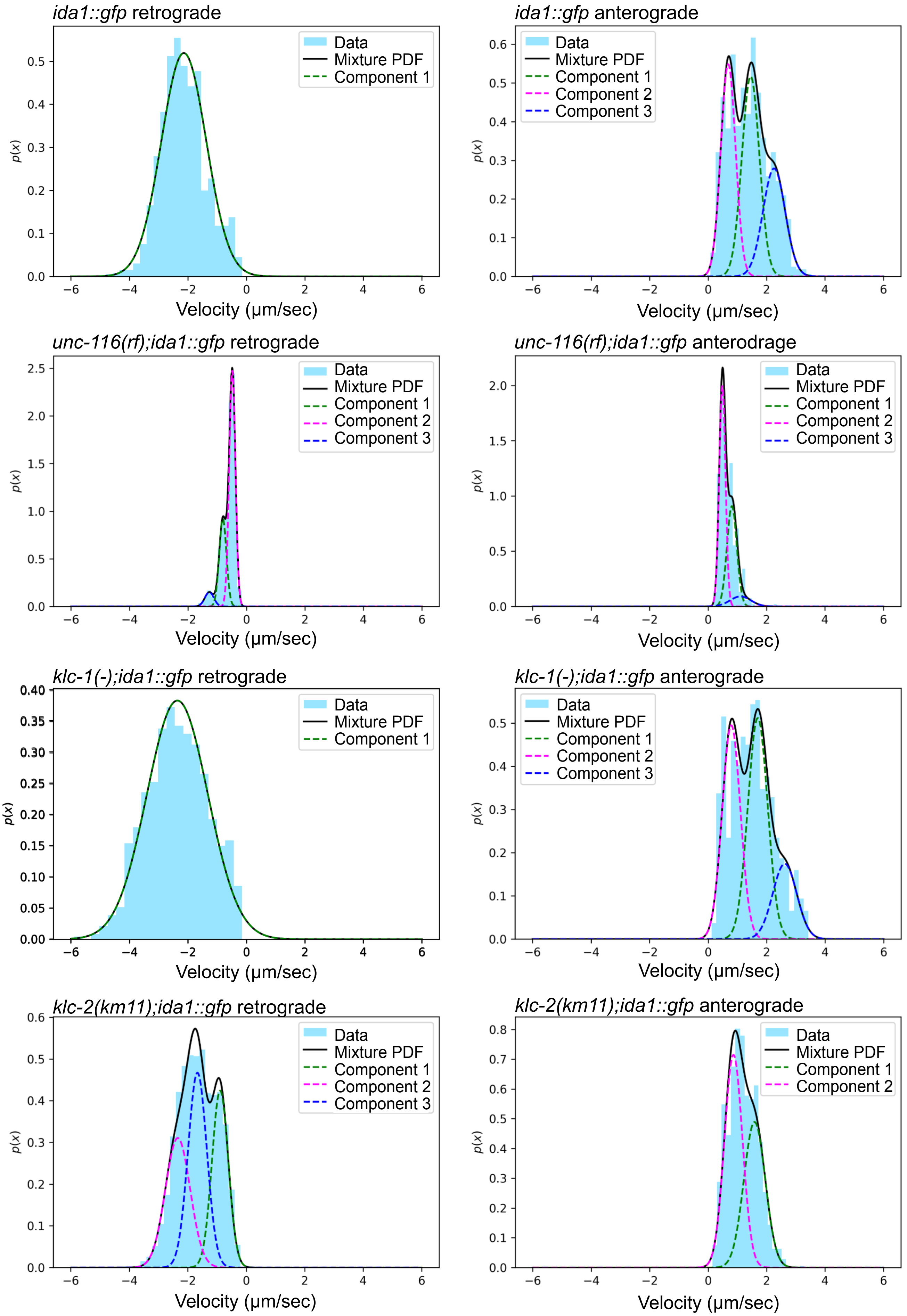

Fig. S4. Gaussian Mixture Models of the DCV velocity distributions.
The data presented in Fig. 4 were fitted with Gaussian Mixture Models (GMM) using the Akaike Information Criterion to determine the optimal number of components for the GMM.

Journal of Cell Science • Supplementary information

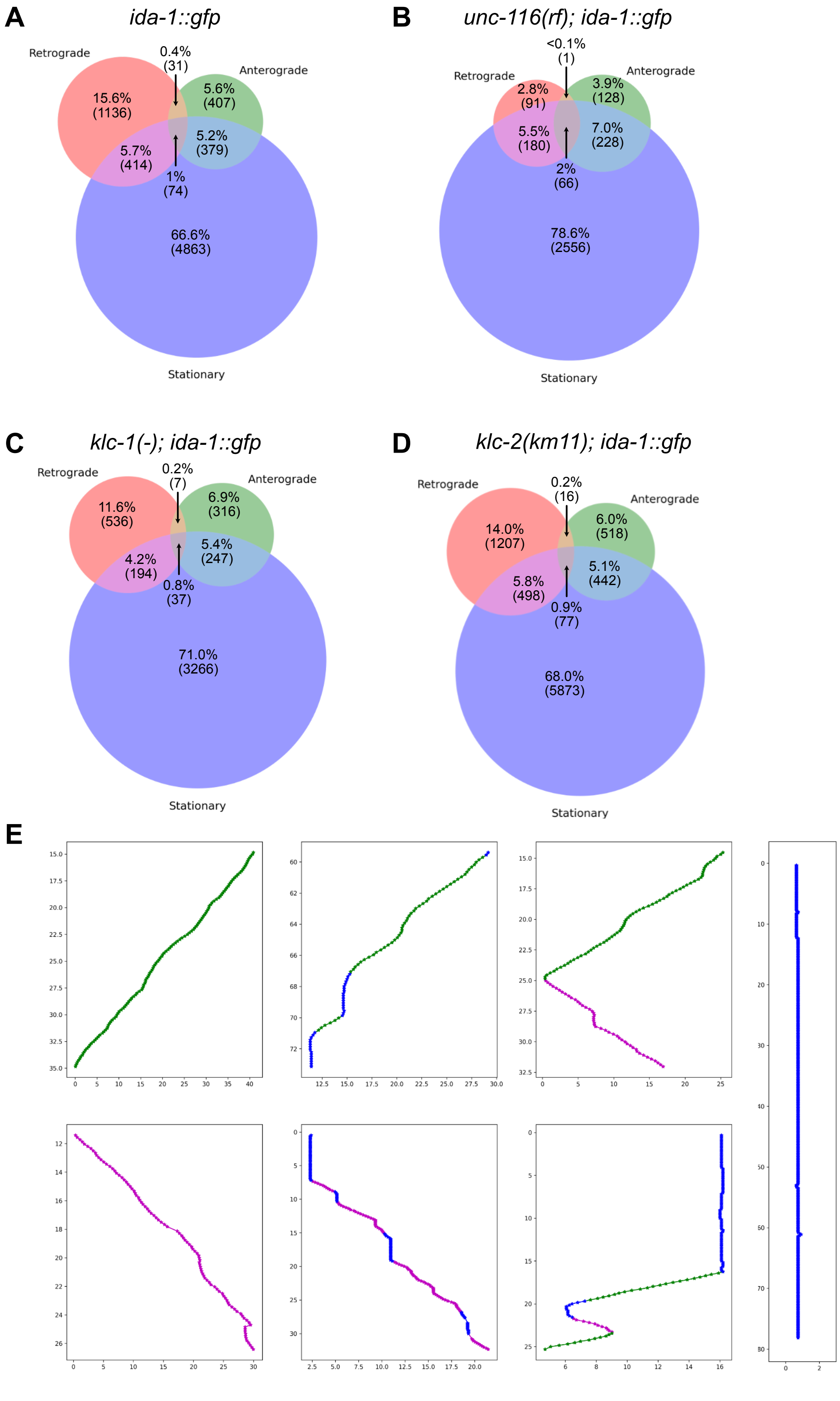

Fig. S5. Analysis of DCV motility.

The proportion of tracks with retrograde, anterograde and stationary segments for each of the four strains, displayed as Venn diagrams. A) *ida-1::gfp*, B) *klc-1(-); ida-1::gfp*, C) *klc-2(rf); ida-1::gfp*, and D) *unc-116(rf); ida-1::gfp*. E) Example *ida-1::gfp* tracks showing different types of movement: retrograde segments are shown in green, anterograde in magenta and stationary segments are in blue. Seven types of tracks include: only retrograde; retrograde and stationary; retrograde and anterograde; only anterograde; anterograde and stationary; retrograde, anterograde and stationary; only stationary. Each track can have multiple segments of each type.

Journal of Cell Science • Supplementary information

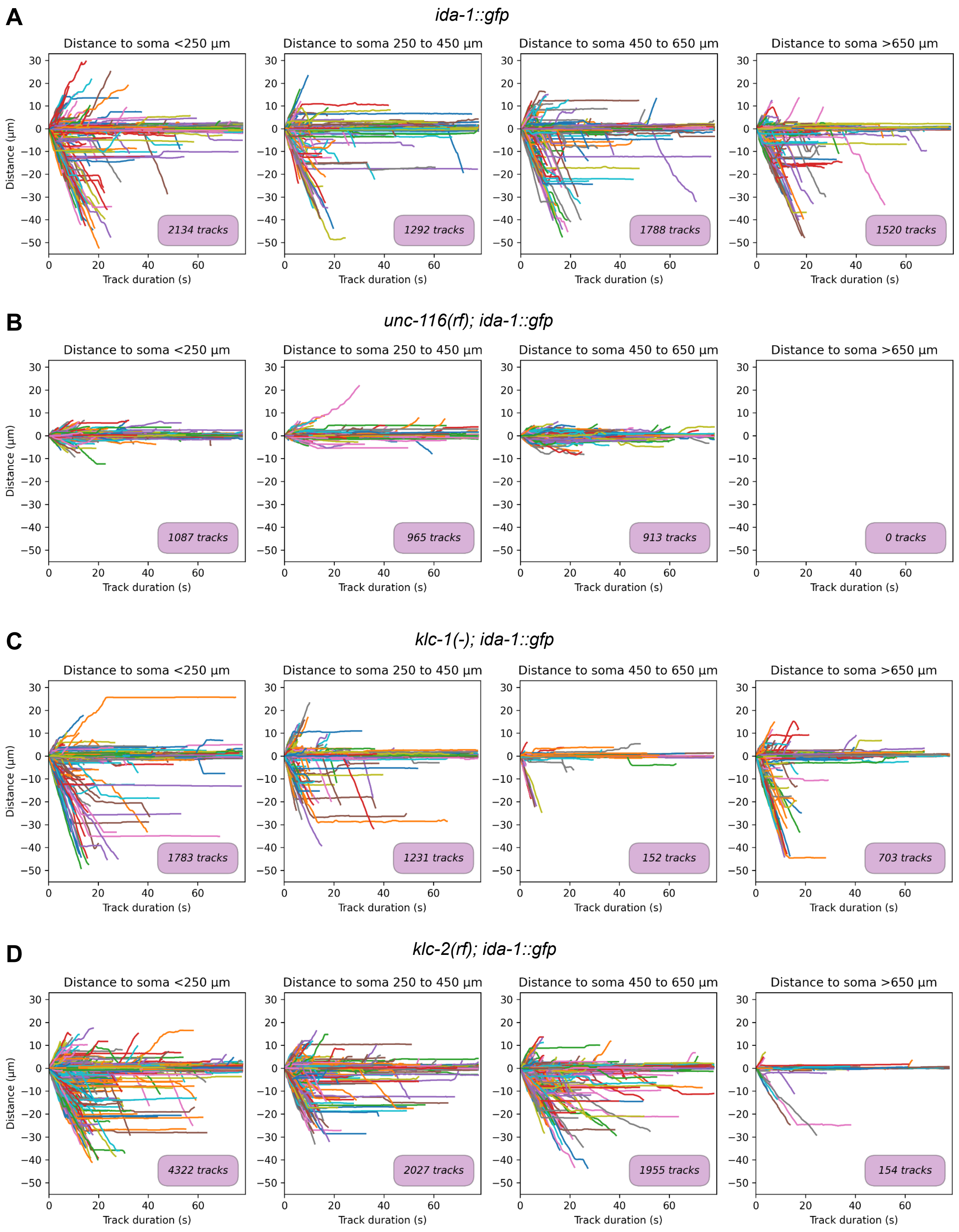

Fig. S6. Individual DCV tracks from different regions in the ALA neuron.

The displacement data (distance moved vs time) for DCVs (as shown in Fig. 3) are divided up according to the position along the ALA neuron that the data was collected and replotted. A) *ida-1::gfp*, B) *unc-116(rf); ida-1::gfp, C) klc-1(-); ida-1::gfp*, and D) *klc-2(rf); ida-1::gfp*. The number of track segments is shown in the insets.

Journal of Cell Science • Supplementary information

Table S1. Statistical analysis of worm swimming. P-values from a One-way ANOVA test (p-value = 1.288e-143) followed by a Tukey-Kramer's HSD test to identify which pairs of group mean body bends per second (see Fig. 5A) were significantly different. The number of worms analysed is shown in the first row. Non-significant comparisons are shaded grey.

Strain	N2	*ida-1:: gfp*	*klc-1(-)*	*klc-1(-); ida-1:: gfp*	*klc-2(km11)*	*klc-2(km11); ida-1:: gfp*	*unc116(rf)*	*unc-116(rf); ida-1::gfp*
Number (n)	49	49	53	51	30	49	38	43
N2		>0.99	>0.99	0.92	< e-15	< e-15	< e-15	< e-15
ida-1::gfp	>0.99		>0.99	0.78	< e-15	< e-15	< e-15	< e-15
klc-1(-)	>0.99	>0.99		0.96	< e-15	< e-15	< e-15	< e-15
klc-1(-); ida-1::gfp	0.92	0.78	0.96		< e-15	< e-15	< e-15	< e-15
klc-2(km11)	< e-15	< e-15	< e-15	< e-15		8.6e-5	< e-15	< e-15
klc-2(km11); ida-1::gfp	< e-15	< e-15	< e-15	< e-15	8.6e-5		< e-15	< e-15
unc-116(rf)	< e-15	< e-15	< e-15	< e-15	< e-15	< e-15		>0.99
unc-116(rf); ida-1::gfp	< e-15	< e-15	< e-15	< e-15	< e-15	< e-15	>0.99	

Journal of Cell Science • Supplementary information

J. Cell Sci.: doi:10.1242/jcs.262148: Supplementary information

Table S2. Statistical analysis of worm crawling. P-values from a One-way ANOVA test (p-value = 4.204e-149) followed by a Tukey-Kramer's HSD test to identify which pairs of mean crawling speeds (see Fig. 5B) were significantly different. The number of worms analysed is shown in the first row. Non-significant comparisons are shaded grey.

Strain	N2	*ida-1:: gfp*	*klc-1(-)*	*klc-1(-); ida-1:: gfp*	*klc-2(km11)*	*klc-2(km11); ida-1:: gfp*	*unc116(rf)*	*unc-116(rf); ida-1::gfp*
Number (n)	29	33	65	49	71	67	55	72
N2		>0.99	3.1e-11	4.9e-4	< e-15	< e-15	< e-15	< e-15
ida-1::gfp	>0.99		5.5e-11	0.0012	< e-15	< e-15	< e-15	< e-15
klc-1(-)	3.1e-11	5.5e-11		0.024	< e-15	1.15e-5	< e-15	< e-15
klc-1(-); ida-1::gfp	4.9e-4	0.0012	0.024		< e-15	2.8e-13	< e-15	< e-15
klc-2(km11)	< e-15	< e-15	< e-15	< e-15		1.04e-6	< e-15	< e-15
klc-2(km11); ida-1::gfp	< e-15	< e-15	1.15e-5	2.8e-13	1.04e-6		< e-15	< e-15
unc-116(rf)	< e-15	< e-15	< e-15	< e-15	< e-15	< e-15		>0.99
unc-116(rf); ida-1::gfp	< e-15	< e-15	< e-15	< e-15	< e-15	< e-15	>0.99	

Table S3. Statistical analysis of worm sensitivity to aldicarb and levamisole. P-values from a Log-Rank test with Bonferroni's correction between the survival functions for each pair of strains from Fig. 5C and D. The null hypothesis was that there was no difference in survival functions. Non-significant comparisons are shown with grey shading. The experiments were repeated independently four times for aldicarb and three times for levamisole. The total number of worms analysed (n) is shown.

	Strain	N2	*ida-1::gfp*	*klc-1(-)*	*klc-2(km11)*	*klc-1(-); ida-1:: gfp*	*klc-2(km11); ida-1:: gfp*
Aldicarb sensitivity	N2 (n=124)		1	< e-10	0.72	0.047	0.036
	ida-1::gfp (n=120)	1		< e-10	0.63	0.43	0.18
	klc-1(-) (n=118)	< e-10	< e-10		< e-10	< e-10	< e-10
	klc-2(km11) (n=120)	0.72	0.63	< e-10		0.0022	1
	klc-1(-); ida-1::gfp (n=120)	0.047	0.43	< e-10	0.0022		4.5e-5
	klc-2(km11); ida-1::gfp (n=120)	0.036	0.18	< e-10	1	4.5e-5	
Levamisole sens.	N2 (n=182)		< e-10	1	< e-10	< e-10	< e-10
	ida-1::gfp (n=176)	< e-10		< e-10	< e-10	0.0508	< e-10
	klc-1(-) (n=180)	1	< e-10		< e-10	2.1e-8	< e-10
	klc-2(km11) (n=176)	< e-10	< e-10	< e-10		< e-10	0.281
	klc-1(-); ida-1::gfp (n=173)	< e-10	0.0508	2.1e-8	< e-10		< e-10
	klc-2(km11); ida-1::gfp (n=178)	< e-10	< e-10	< e-10	0.281	< e-10	

Table S4. Statistical analysis of worm lifespan data. P-values from a Log-Rank test with Bonferroni's correction between the survival functions for each pair of strains from Fig. 6. The null hypothesis was that there was no difference in survival functions. Non-significant comparisons are shown with grey shading.

Strain	N2	*ida-1::gfp*	*klc-1(-)*	*klc-2(km11)*	*unc-116(rf)*	*klc-1(-); ida-1:: gfp*	*klc-2(km11); ida-1:: gfp*	*unc-116(rf); ida-1::gfp*
N2		2e-4	1	1	< e-10	< e-10	1.0e-4	< e-10
ida-1::gfp	2e-4		1.0e-4	7.0e-4	< e-10	< e-10	< e-10	< e-10
klc-1(-)	1	1.0e-4		1	< e-10	< e-10	7.0e-4	< e-10
klc-2(km11)	1	7.0e-4	1		< e-10	< e-10	3.0e-5	< e-10
unc-116(rf)	< e-10	< e-10	< e-10	< e-10		< e-10	< e-10	0.364
klc-1(-); ida-1::gfp	< e-10	< e-10	< e-10	< e-10	< e-10		0.14	< e-10
klc-2(km11); ida-1::gfp	1.0e-4	< e-10	7.0e-4	3.0e-5	< e-10	0.14		< e-10
unc-116(rf); ida-1::gfp	< e-10	< e-10	< e-10	< e-10	0.364	< e-10	< e-10	

Table S5. *C. elegans* strains used and generated in this study

Strain	Referred to here as	Genotype	Source
BL5752	ida-1::gfp	*inIs182 [ida-1p::ida-1::gfp] I; inIs181 [ida-1p::ida-1::gfp] IV*	(Zahn et al., 2004)
RB1975	*klc-1(-)*	*klc-1(ok2609) IV*	CGC
KU801	*klc-2(km11)*	*klc-2(km11) V*	CGC
	unc-116(rf)	*unc-116(rh24sb79) III*	(Yang et al., 2005)
OL0296	*klc-1(-); ida-1::gfp*	*inIs182 [ida-1p::ida-1::gfp] I; inIs181 [ida-1p::ida-1::gfp] IV; klc-1(ok2609) IV*	This study
OL0340	*klc-2(km11); ida-1::gfp*	*inIs182 [ida-1p::ida-1::gfp] I; inIs181 [ida-1p::ida-1::gfp] IV; klc-2(km11) V*	This study
OL0358	*unc-116 (rf); ida-1::gfp*	*inIs182 [ida-1p::ida-1::gfp] I; inIs181 [ida-1p::ida-1::gfp] IV; unc-116(rh24sb79) III*	This study

Journal of Cell Science • Supplementary information

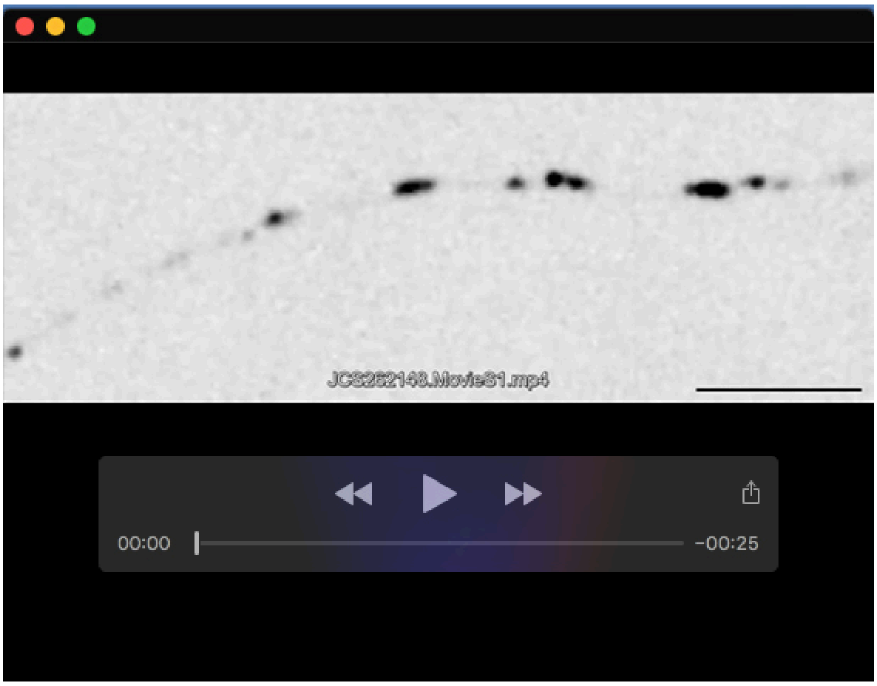

Movie 1. Wild-type DCV movement. A spinning disk confocal video of DCV movement in the ALA neuron in an *ida-1::gfp* worm. The nerve terminal (microtubule plus ends) is on the right. Kinesin-driven DCVs correspond to particles moving to the right, with dynein-driven DCVs moving to the left. The frame rate is 2 times real time. Scale bar = 5 µm.

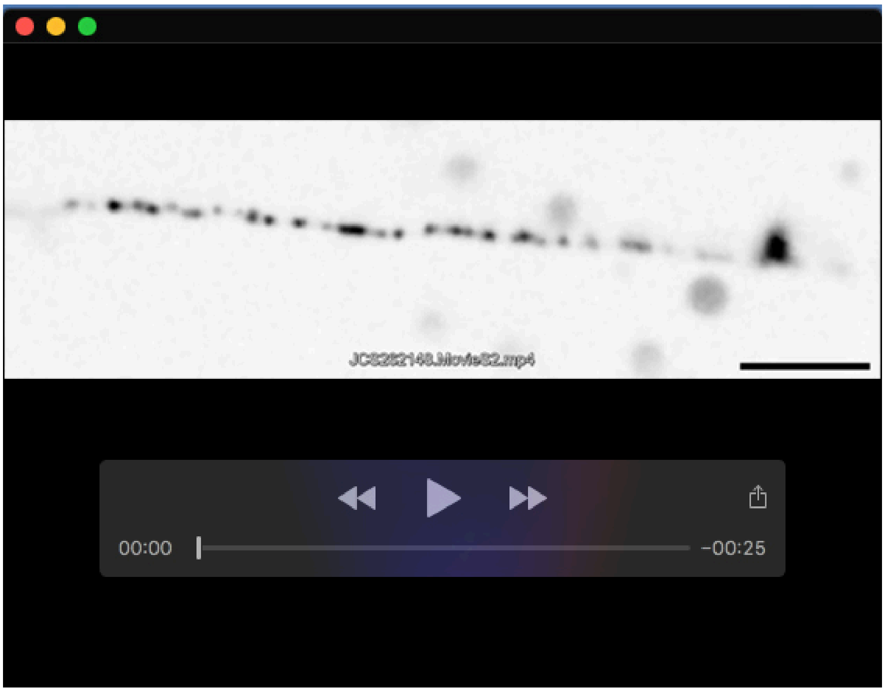

Movie 2. DCV movement in the *unc-116(rf)* mutant. A spinning disk confocal video of DCV movement in the ALA neuron in a *unc-116(rf); ida-1::gfp* worm. The nerve terminal (microtubule plus ends) is on the right. Kinesin-driven DCVs correspond to particles moving to the right, with dynein-driven DCVs moving to the left. The frame rate is 2 times real time. Scale bar = 5 µm.

Journal of Cell Science • Supplementary information

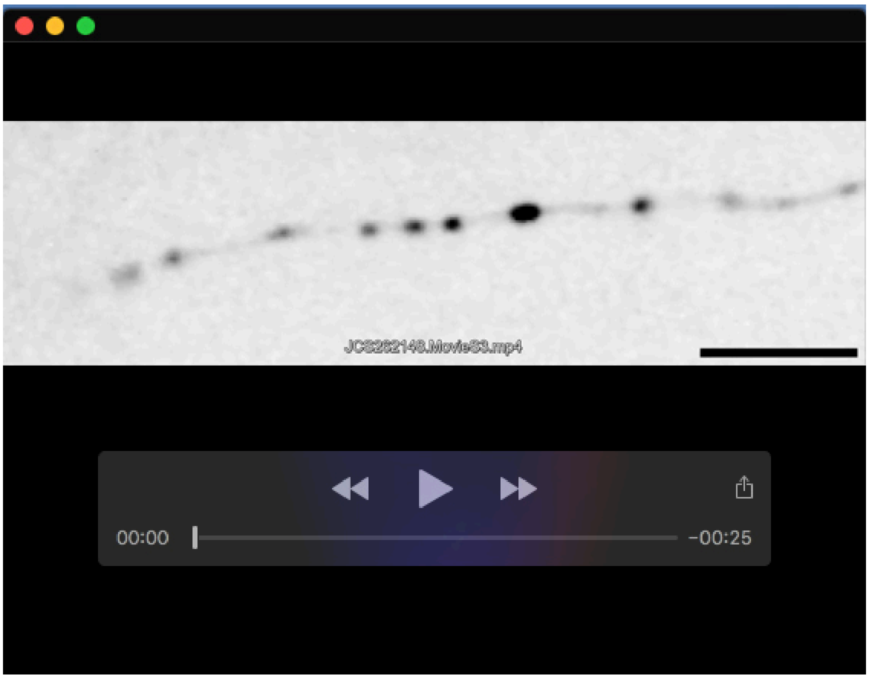

Movie 3. DCV movement in the *klc-1(-) mutant*. A spinning disk confocal video of DCV movement in the ALA neuron in a *klc-1(-); ida-1::gfp* worm. The nerve terminal (microtubule plus ends) is on the right. Kinesin-driven DCVs correspond to particles moving to the right, with dynein-driven DCVs moving to the left. The frame rate is 2 times real time. Scale bar = 5 μm.

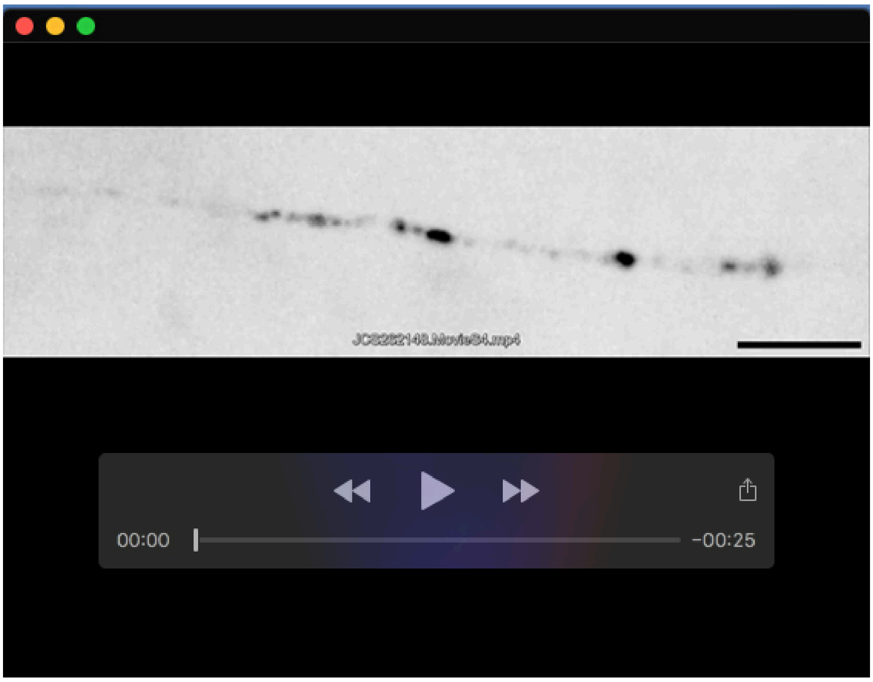

Movie 4. DCV movement in the *klc-2(km11) mutant*. A spinning disk confocal video of DCV movement in the ALA neuron in a *klc-2(km11); ida-1::gfp* worm. The nerve terminal (microtubule plus ends) is on the right. Kinesin-driven DCVs correspond to particles moving to the right, with dynein-driven DCVs moving to the left. The frame rate is 2 times real time. Scale bar = 5 μm.

Journal of Cell Science • Supplementary information
