# Supplementary material for: The role of kinesin-1 in neuronal dense core vesicle transport, locomotion and lifespan regulation in C. elegans
Source: J Cell Sci. 2024 Sep 6;137(17):jcs262148. doi: 10.1242/jcs.262148 (PMC11423817; doi:10.1242/jcs.262148)
Supplement: Supplementary file 1 [file joces-137-262148_review_history.pdf]

The role of Kinesin-1 in neuronal dense core vesicle transport, locomotion and lifespan regulation in *C. elegans*

Anna Gavrilova, Astrid Boström, Nickolay Korabel, Gino B. Poulin, Sergei Fedotov and Victoria J Allan
DOI: 10.1242/jcs.262148

Editor: Giampietro Schiavo

Review timeline

Original submission:	25 March 2024
Editorial decision:	30 April 2024
First revision received:	9 August 2024
Accepted:	9 August 2024

Original submission

First decision letter

MS ID#: JOCES/2024/262148

MS TITLE: The role of Kinesin-1 in neuronal dense core vesicle transport and lifespan regulation in C. elegans

AUTHORS: Anna Gavrilova, Astrid Boström, Nickolay Korabel, Gino B Poulin, Sergei Fedotov, and Victoria J Allan

We have now reached a decision on the above manuscript.

To see the reviewers' reports and a copy of this decision letter, please go to: https://submit-jcs.biologists.org and click on the 'Manuscripts with Decisions' queue in the Author Area. (Corresponding author only has access to reviews.)

As you will see, the reviewers raised several criticisms preventing me from accepting the paper at this stage. They suggest, however, that a revised version might prove acceptable, if you can address at least in part their concerns. If you think that you can deal satisfactorily with the criticisms on revision, I would be pleased to see a revised manuscript. I am happy to be contacted if you have a specific plan for revision or if you wish to discuss its logistic.

Please ensure that you clearly highlight all changes made in the revised manuscript. Please avoid using 'Tracked changes' in Word files as these are lost in PDF conversion.

I would be grateful if you would also provide a point-by-point response detailing how you have dealt with the points raised by the reviewers in the 'Response to Reviewers' box. Please attend to all of the reviewers' comments. If you do not agree with any of their criticisms or suggestions please explain clearly why this is so.

Reviewer 1

Advance summary and potential significance to field

© 2024. Published by The Company of Biologists under the terms of the Creative Commons Attribution License (https://creativecommons.org/licenses/by/4.0/).

While DCVs are known to be transported by kinesin-3, the authors revealed an additional role of kinesin-1 on DCV transport. Especially, KLC-1 and KLC-2 seem to play different roles in regulating DCV transport. Further, kinesin-1 affects worm bends and life span. My biggest concern is the lack of statistics. There are no group comparisons using t-tests or ANOVA. Therefore, the significance of this study can be questioned. Without proper statistic comparing the different experimental groups this paper cannot be properly evaluated by any peer-reviewer.

Comments for the author

Major concerns:
=> It is surprising that klc-2 had an effect on anterograde direction while klc-1 not. Both light chains seemed to be of similar structure and function. This needs to better discussed or experimentally evaluated. What is the homology between KLC-1/KLC-2 and mammalian KLC? What are the sequence differences between KLC-1 and KLC-2? What would be the purpose to encode KLC in two genes? Is there any literature about different functions of KLC-1 and KLC-2?
=> How to explain reduced thrashing in the mutants? If thrashing assay shows significant differences in the mutants then we may assume motor neuron and muscle cell functions being affected. It would be also interesting to see if mechanosensation in worms is affected. For both nose touch as well as body touch (mec-response) and if the worm still can discriminate between harsh and soft touch. For these assays standard protocols are available.
=> What is the relation between reduced body bends and reduced lifespan? How to explain reduced body bends and reduced life span in relation to dysfunctional kinesin-1? Such as kinesin-1 is the major transporter of mitochondria. Is oxygen consumption affected in worms? ROS increased? Methods exist to measure ROS and oxygen consumption in worms.
=> If KLC-1 and KLC-2 have different effects on DCV transport do they perhaps also have different effects on mitochondria transport? These experiments would also underscore the specificity of the findings.
=> My major problem is the quantification of motility data. All data are represented way to descriptive. From kymographs many data can be nicely extracted and properly blotted in graphs with proper statistics. Examples are velocity, pausing, run lengths and directional changes. Then proper statistics can be done using ANOVA or t-tests. I strongly suggest the authors to look at papers from Kang Shen-lab, Koushika-lab or Wagner-lab how they blot and quantify data.
=> Since the authors found a “synergistical” relation between klc mutants and ida-1::gfp it is critical to investigate the functional relation between KLC and IDA-1 otherwise this manuscript just scratches the surface of something interesting. Authors can employ methods such as co-localization, BiFC and Co-IPs to investigate functional interactions between KLC and IDA-1. Also if ida-1::GFP extends lifespan in klc mutants, IDA-1 may rescue the effect of transport imbalances in klc mutants. Thus, IDA-1 may need to be overexpressed (not using the integrated strain) in klc mutants and transport parameters measured. Vice versa, creation of an IDA-1::GFP rescue worm (such as ida-1(ok409);ida::gfp) is recommended to see if such synergistical effects still exist.
=> Another concern is the novelty. The role of kinesin-1 in dense core vesicle transport has been already shown by others (e.g., Lim et al., 2017, MBC; Iacobucci & Gunawardena, 2018 J Neurochem; Varadi et al., 2003, BBRC).
Specifically, the authors mention a model in which kinesin-3 drives DCVs out of the cell body (at fast speeds) with kinesin-1 then taking over (at lower speeds)
which needs further validation. What are the DCV transport speeds of kinesin-1 in different regions of the axon such as soma, initial axonal segment, proximal and distal axonal areas? What are the speeds of UNC-104 mediated transport of DCVs in these areas? Particularly, the authors claim in the Introduction: “Here we show that kinesin-1 is the second motor for this DCV transport in C. legans.” Though I am not happy with this terminology (“second motor”) I wonder how exactly are the functions of the “first motor” (kinesin-3) and the second motor (kinesin-1) discriminated from each other? Again, the authors just seem to scratch the surface of something interesting and with the same tools in their hands the study can be advanced to a much deeper understanding by investing just a little bit more time.
=> Literature can be cut down. This is not a review. For example Introduction:
The sentence “More than one kinesin type can transport the same cargo” is followed by 10 citations.

Minor Concerns:
Abstract

© 2024. Published by The Company of Biologists under the terms of the Creative Commons Attribution License (https://creativecommons.org/licenses/by/4.0/).

- “reduced the frequency of body bends during swimming” = reduced the frequency of body bends in thrashing assays
- “We also determined lifespan, finding that klc-1(-) or klc-2(rf) single mutants were wild-type…” => “were wild-type” = resembled wild-type life span
- “…whereas the unc-116(rf), ida-1::gfp and unc-116(rf); ida-1::gfp strains were short-lived.” => what is the difference between ‘unc-116(rf), ida-1::gfp’ and ‘unc-116(rf); ida-1::gfp’?
- “Strikingly, the ida-1::gfp transgenic…” = Strikingly, the ida-1::gfp transgenic animal…

Introduction
- “In addition to relying on standard diffusion…” => what is “standard diffusion”? Molecular motors are either processive on non-processive. There is a skating (dynein) or “biased diffusion” phenomenon but this needs to be explicitly explained (if applicable)
- “While the klc-1 and -2 mutant worms had normal longevity” = While the klc-1 and -2 mutant worms revealed wildtype longevity
- “… but combining ida-1::gfp with either klc mutation gave animals with a longer lifespan than wildtype worms or either parental strain” => this sentence needs a complete overhaul

Results
- “The ALA neuron is ideal for imaging, as it extends two axons down each side of the worm from the cell body in the head” => a pictographic representation of the ALA is recommended especially for the readers with less C. elegans expertise.
- All strains need to better explained (defined). What is the resulting gene product of rh24sb79, km11 and ok2609? How large are the deletions? Are there frameshifts of premature stops? If knockout (ok2609) is there any literature evidence showing a Western to proof for knockout? If not authors need to do it.
- “Inappropriate branching of axons has been observed in zebrafish (Auer et al., 2015), but it has not been reported in C. elegans kinesin-1 mutants to our knowledge.” => The authors seem to make a point on the branching, thus it shall be quantified using existing image analysis tools.
- Page 5: “…we imaged single z-planes by spinning disk confocal microscopy” = we imaged single z-planes of ALA neurons by spinning disk confocal microscopy
- Did the authors used levamisole to immobilize the worms? If yes, it needs to be mentioned because it has been reported to affect transport (Koushika lab).
- Page 6: “In addition, there were notable spots where particles clustered together and did not move, which likely correspond to the periodic swellings observed by EM along the length of the ALA neuron that contain clusters of DCV some of which may occur at en passant synapses” => needs quantification
- Page 6: “When approaching such a cluster, the moving DCV could either stop or pass through. Clusters were sometimes seen to shed a DCV that then moved off in either direction.” => needs quantification
- Table 1: Groups need to be compared using ANOVA to evaluate statistical differences.
- “Comparison of the distribution of the individual segment velocities showed that both directions were significantly different to those in wild-type ida-
1::gfp worms” => I don’t see any statistics.
- “In contrast to the klc-2(rf) mutant, the complete loss of klc-1 had only a small, statistically insignificant effect on plus-end-directed mean velocity” =>
Once more no statistics comparing groups (t-test or ANOVA) are provided by the authors.
- Page 7: “This clearly shows the greater distance travelled by dynein-driven DCVs within a set time in the klc-1(-) mutant compared to the wild-type and klc-2(rf) mutant.” => Run lengths values can be easily measured and blotted and proper statistic done (once more please refer to papers from established groups).
- Page 7: “swimming” => “thrashing assay” is the preferred term
- Page 7: “indicating neuronal dysfunction” => Aldicarb assays is also a good assay to test for this
- Figure 5: once more proper statistics (such as ANOVA) is missing.
- Page 8: “the bending frequency of both unc-116(rf) and unc-116(rf); ida-1::gfp was extremely low and not significantly different” => it needs a t-test to underscore this
- Page 8: “being somewhat worse than unc-116(rf) alone at later times” =>
scientific writing needs to be improved

<u>Reviewer 2</u>

© 2024. Published by The Company of Biologists under the terms of the Creative Commons Attribution License (https://creativecommons.org/licenses/by/4.0/).

Advance summary and potential significance to field

The molecular mechanism of axonal transport of DCVs is not fully understood. Previous studies have shown the function of the UNC-104 motor protein in the axonal transport of DCVs, but these studies suggest that another molecular motor is involved in this phenomenon. In this paper, Gavriova et al. analyze the axonal transport of DCVs using C. elegans genetics.

The authors used IDA-1 as a marker for DCVs and observed their distribution in the ALA neuron, which has a long axon and is suitable for analyzing axonal transport. They found that the motility of DCVs is affected in unc-116 mutant worms by quantification of the kymographs. Additionally, they noted that the lifespan is affected by the unc-116 mutation and overexpression of IDA-1::GFP.

The authors present genetic evidence that UNC-116 is required for axonal transport. This work will contribute to our understanding of how DCVs are transported by two molecular motors. The analysis is performed well but some discussions are not supported by experimental evidence or citations.

Comments for the author

1. In Abstract, unc-116(rf), klc-2(rf) and klc-1(-). (rf) and (-) are not standard nomenclature. The authors need to clearly define them.

2. It is described that "Gaussian fitting revealed three peaks (Fig. S5), which likely correspond to kinesin-1 (slow), kinesin-3/UNC-104 (fast) and a mixture of the two (intermediate), as has beenpreviously observed for mixtures of kinesin-1 and -3 motors". On the other hand, it is described that " Notably, however, the plus-end-directed velocities in the unc-116(rf) mutant, which still has wild type UNC-104 motors, is drastically slowed to a velocity not seen in normal axons, despite UNC-104 being a fast motor. This may be due to kinesin-3 motors detaching more readily from microtubules than kinesin-1 (Arpag et al., 2019; Arpag et al., 2014; Norris et al.,2014), particularly under load. The presence of the partially functional UNC-116 motor....."

I cannot understand this argument. Please reconsider and rewrite them. If UNC-104 is faster, and the intermediate peak is generated by the cooperation between UNC-104(fast) and UNC-116(slow), unc-116(rf) should show faster movement.
If slower movement in unc-116(rf) is caused by frequent detachment, the run length of DCB should be shorter in unc-116(rf). Is this correct?

A simple explanation is that UNC-104 is slower than UNC-116. Are there any publication that compared the velocity of UNC-104 and UNC-116?

"Alternatively, since dimerizationof kinesin-3 motors has been shown to make them super-processive (Soppina et al., 2014),perhaps UNC-104 is unable to dimerise in the absence of functional UNC-116."
There is no evidence. For instance, synaptic vesicles are transported by UNC-104 without UNC-116. Moreover, if this is case, the velocity of DCVs should not exhibit three peaks. The fastest peak should be generated by UNC-104 only.

"Another possibility there is not enough UNC-104 present on the DCVs to be able to drive their rapid movement through the highly crowded axonal environment without the help of kinesin-1,as has been observed for DCV motility in MRC5 cells (Serra-Marques et al., 2020)."
Again, presented evidence is not consistent with this scenario. If this is true, again, the velocity of DCV should not exhibit three peaks.

3. The authors have suggested that UNC-116 is fundamental for the axonal transport of DCVs. Then, why is the axonal transport of DCVs almost diminished in unc-104 mutants? Could the authors quantify the motility of DCVs (velicity, run length etc.) in unc-104 mutants to show the UNC-116 dependent DCV movement only?

4. "Altogether, these data strongly suggests that kinesins-1 and -3 work in tandem to drive DCV along the ALA neuron"

© 2024. Published by The Company of Biologists under the terms of the Creative Commons Attribution License (https://creativecommons.org/licenses/by/4.0/).

The word "tandem" is ambiguous. Does the authors use the word to say "cooperative" or "redundant"?
Or does the authors use the word "tandem" to say that UNC-104 work in the exit from the cell body and UNC-
116 work in the axon?
5. Plus-end-directed movement was less processive and had an average velocity of 1.4 ± 0.68 ?m/s (Table 1).
(1) The word "processive" is used in enzymology to describe the behavior of enzymes, such as kinesin, that move along a substrate without detaching. While "processive" is used in the context of kinesin to represent "run length", its use to describe the run length of vesicles is not appropriate.

(2) Please show the run length in Table S1.

Reviewer 3

Advance summary and potential significance to field

Gavrilova et al. explore the role of kinesin-1 proteins in DCV transport in ALA neurons in C.Elegans. They take advantage of strains expressing a DCV marker ida-1::gfp in various kinesin-1 mutant backgrounds that target different subunits of the complex. They observe different and in some cases unexpected phenotypes depending on which subunit of the kinesin-1 complex is disrupted. A unc-116 reduced function mutation (in the kinesin heavy chains) and klc-2 (one of the kinesin light chains) reduce DCV transport in both retrograde and anterograde transport. This translates to defects in worm motility. Perhaps surprisingly, a klc-1 mutation didn't affect anterograde transport, but did impact on dynein mediated retrograde transport. Together, these findings implicate kinesin-1 proteins in DCV motility in C.Elegans, as in several other species. Most interestingly, this deregulation of transport appears to have some positive effects on worm health and lifespan because, whilst klc-1 and klc-2 mutations alone did not have a phenotype, there was an effect when combined with the ida1::gfp transgene. Thus, in this genetic background, kinesin-1 can impact on lifespan.

These findings are novel, the experiments are well performed and quantified, and appear robust, and are not overstated. It is still unclear what the mechanistic basis for these interesting observations is but this is probably for future work. I am not an expert in C.Elegans genetics, neuronal biology or aging, so it is difficult for me to provide critical evaluation of this (very significant) part of the work. Hopefully, this will come from another reviewer. However, from the perspective of better defining the role of kinesin-1 proteins in DCV transport in worms, and data pointing to impacts on health/lifespan, this work will be valuable and is of general interest.

Comments for the author

My main comment for improvement of the work is in presentation. For a conceptually straightforward paper, I thought that is overly long and somewhat heavy going. In particular, there is a large amount of discussion and citation of other literature in the results, that I think distracts from the author’s findings and the clarity of their presentation. I would also suggest some attempt is made to condense the discussion to focus on the key findings here.

First revision

Author response to reviewers' comments

Response to Reviewers

We thank the reviewers for their comments, to which we respond in detail below. We would recommend that the reviewers look at the formatted version of these comments, which is provided as a supplemental file, as all formatting is lost when text is pasted into the web portal.

© 2024. Published by The Company of Biologists under the terms of the Creative Commons Attribution License (https://creativecommons.org/licenses/by/4.0/).

We have modified the text substantially, and would like to explain that a document showing the main text insertions or relocations (but not deletions, to avoid complexity) is provided as a supplemental document. The main text submission is therefore a 'clean' version, submitted with approval from the JCS office.

Reviewer 1 Advance Summary and Potential Significance to Field...
While DCVs are known to be transported by kinesin-3, the authors revealed an additional role of kinesin-1 on DCV transport. Especially, KLC-1 and KLC-2 seem to play different roles in regulating DCV transport. Further, kinesin-1 affects worm bends and life span. My biggest concern is the lack of statistics. There are no group comparisons using t-tests or ANOVA. Therefore, the significance of this study can be questioned. Without proper statistic comparing the different experimental groups this paper cannot be properly evaluated by any peer-reviewer.
Response: Full statistical analysis was provided in the original submission as supplemental tables 1-3, which were referred to in each relevant figure legend and in the text. Key statistics were also included on figure 4 and described in the methods. However, we realise that they were clearly easily missed. We have therefore included statistical analysis in table 1, and key statistics are provided in the text. Since many of the comparisons are complex due to the number of strains analysed, full statistics are provided in tables S1-S4, which are referred to in the text and the figure legends.

Reviewer 1 Comments for the Author...
Major concerns:
=> It is surprising that klc-2 had an effect on anterograde direction while klc-1 not. Both light chains seemed to be of similar structure and function. This needs to better discussed or experimentally evaluated. What is the homology between KLC-1/KLC-2 and mammalian KLC? What are the sequence differences between KLC-1 and KLC-2? What would be the purpose to encode KLC in two genes? Is there any literature about different functions of KLC-1 and KLC-2?
Response: We realise that we did not discuss this in the original submission, which was an omission. We now include text in the introduction as well as a figure (Fig. S1) showing sequence comparisons of C. elegans KLC-1 and -2, and a comparison of them with human KLC-1 and KLC-2 (please note that the gene numbering does not imply similarity, as worm KLC-2 is more similar to both human KLCs than worm KLC-1 is). The new data we have included provides clear evidence that KLCs -1 and -2 in worms are not completely interchangeable, even though they can probably both transport the same cargo in many cases. The role of different KLCs in any organism is an important question that is not yet resolved. Hence this work will be of great interest to the kinesin-1 field.
=> How to explain reduced thrashing in the mutants? If thrashing assay shows significant differences in the mutants then we may assume motor neuron and muscle cell functions being affected. It would be also interesting to see if mechanosensation in worms is affected. For both nose touch as well as body touch (mec-response) and if the worm still can discriminate between harsh and soft touch. For these assays standard protocols are available.
Response: We thank the referee for prompting us to look into this question further. Rather than use the mechanosensation assays mentioned, we used aldicarb (as also suggested by this reviewer) and levamisole treatment to identify whether pre-synaptic or post-synaptic function at neuromuscular junctions (NMJs) was compromised. This revealed an unexpected and clear difference between the klc-1 and klc-2 mutants, with the former being aldicarb resistant and the latter being hypersensitive to levamisole. By comparison with the literature on aldicarb and levamisole sensitivity, the klc-2 response is likely due to defects in glutamate receptor subunit trafficking that are seen in the AVA command interneuron in the same klc-2 mutant (Hoerndli et al., 2013 and 2015), since GluR mutants are also levamisole sensitive (Sadananda & Subramaniam, 2021). The klc-1 mutant aldicarb resistance is likely due to defects in dense core vesicle trafficking in motor neurons and command interneurons, but this remains to be tested. Altogether, these new data allow us to present a much clearer interpretation of the roles of both KLCs.
=> What is the relation between reduced body bends and reduced lifespan? How to explain reduced body bends and reduced life span in relation to dysfunctional kinesin-1? Such as kinesin-1 is the major transporter of mitochondria. Is oxygen consumption affected in worms? ROS increased? Methods exist to measure ROS and oxygen consumption in worms.
Response: We have mentioned in the text that one of the cargos that may be important for lifespan is indeed the mitochondria (see also the comment below). However, we think an analysis of mitochondrial respiration is outside the scope of this study.

© 2024. Published by The Company of Biologists under the terms of the Creative Commons Attribution License (https://creativecommons.org/licenses/by/4.0/).

=> If KLC-1 and KLC-2 have different effects on DCV transport do they perhaps also have different effects on mitochondria transport? These experiments would also underscore the specificity of the findings.
Response: This is a complicated question, since there is conflicting data about whether mitochondrial transport involves both KLCs or only one of them, as now discussed in the text. One likely explanation for this complexity is simply that there are tissue- or cell type-specific differences between klc-1 expression levels. Once the key neurons or cell types involved in the responses we report have been identified, then effects on mitochondrial transport will be a crucial topic for further investigation.
=> My major problem is the quantification of motility data. All data are represented way to descriptive. From kymographs many data can be nicely extracted and properly blotted in graphs with proper statistics. Examples are velocity, pausing, run lengths and directional changes. Then proper statistics can be done using ANOVA or t-tests. I strongly suggest the authors to look at papers from Kang Shen-lab, Koushika-lab or Wagner-lab how they blot and quantify data.
Response: We would like to stress that our analysis uses sophisticated automatic annotation of kymographs using KymoButler, followed by sliding window segmentation of the KymoButler tracks using custom-written Python scripts. This builds on our expertise in analysis of endocytic organelle motility in cultured cells (e.g. Han et al., 2020, eLife 9, e52224; Flores-Rodriguez et al., 2011, PLoS ONE 6, e24479). This segmentation provides a wealth of data to analyse. Our figure 4 provides full information on the velocity of movement, and statistical analysis (by Kolmogorov-Smirnov test: now included in Table 1 as well as shown on the figure as before) of the anterograde and retrograde motility in each strain. We had indeed quantified directional changes and the number of movements in each direction, as presented in Figure S3 (now S5). It is not possible to analyse run lengths in our data because for this to be accurate, both the start and the end of a run must be obvious, but a large proportion of the DCVs imaged were moving as the entered or left one side of the field of view, or moved right across the field without stopping.
=> Since the authors found a “synergistical” relation between klc mutants and ida-1::gfp it is critical to investigate the functional relation between KLC and IDA-1 otherwise this manuscript just scratches the surface of something interesting. Authors can employ methods such as co-localization, BiFC and Co-IPs to investigate functional interactions between KLC and IDA-1. Also if ida-1::GFP extends lifespan in klc mutants, IDA-1 may rescue the effect of transport imbalances in klc mutants. Thus, IDA-1 may need to be overexpressed (not using the integrated strain) in klc mutants and transport parameters measured. Vice versa, creation of an IDA-1::GFP rescue worm (such as ida-1(ok409);ida::gfp) is recommended to see if such synergistical effects still exist.
Response: We are using IDA-1::GFP as a marker for DCVs. We are not suggesting that it interacts directly with kinesin-1 (via either KLCs or the heavy chain) and so these tests, although potentially interesting, would not add to the current work. The interplay we talk about is between the number and transport of DCVs, with the former being influenced by IDA-1 expression levels, and the latter being due to the combination of kinesin-1, kinesin-2 and dynein. We have modified the text to clarify what we mean by the interplay between kinesin-1 and IDA-1.
=> Another concern is the novelty. The role of kinesin-1 in dense core vesicle transport has been already shown by others (e.g., Lim et al., 2017, MBC; Iacobucci & Gunawardena, 2018 J Neurochem; Varadi et al., 2003, BBRC). Specifically, the authors mention a model in which kinesin-3 drives DCVs out of the cell body (at fast speeds) with kinesin-1 then taking over (at lower speeds) which needs further validation. What are the DCV transport speeds of kinesin-1 in different regions of the axon such as soma, initial axonal segment, proximal and distal axonal areas? What are the speeds of UNC-104 mediated transport of DCVs in these areas? Particularly, the authors claim in the Introduction: “Here, we show that kinesin-1 is the second motor for this DCV transport in C. elegans.” Though I am not happy with this terminology (“second motor”) I wonder how exactly are the functions of the “first motor” (kinesin-3) and the second motor (kinesin-1) discriminated from each other? Again, the authors just seem to scratch the surface of something interesting and with the same tools in their hands the study can be advanced to a much deeper understanding by investing just a little bit more time.
Response: We acknowledge that there have been other studies on this topic, as we describe in the introduction and discussion. However, this has not been shown in C. elegans before, and all of the mammalian studies have been performed in cell lines. The speed of motion of the different motors is covered in general in the discussion, but it is a tricky issue to determine speeds using mutants. This is a question that is ripe for further study using auxin-mediated protein degradation, which we feel is the approach needed to clarify how the two kinesins work together. We have removed the “second” from the text. We addressed the question of whether position along the ALA neuron was

© 2024. Published by The Company of Biologists under the terms of the Creative Commons Attribution License (https://creativecommons.org/licenses/by/4.0/).

important in the initial submission, which contained a supplementary figure (S4) that presented the tracks from different regions, showing no obvious differences according to position.
=> Literature can be cut down. This is not a review. For example Introduction: The sentence “More than one kinesin type can transport the same cargo” is followed by 10 citations.
Response: We believe that it is important to give credit to work which has gone before. A particularly good example of how this is helpful to the understanding of motor function is given in Reto Gassman’s recent paper on kinesin-1, dynein and JIP3 in both in vivo in worms and using purified proteins (Celestino et al., 2022, J. Cell Biol. 221, e202110057). However, we have chosen the most relevant paper from any individual lab (rather than citing more than one) to reduce the number of citations.
Minor Concerns:
Abstract
Response: The abstract has been substantially revised and the text highlighted in the first 3 points below is no longer present. The final point has been corrected.
- “reduced the frequency of body bends during swimming” = reduced the frequency of body bends in thrashing assays
- “We also determined lifespan, finding that klc-1(-) or klc-2(rf) single mutants were wild-type…” => “were wild-type” = resembled wild-type life span
- “…whereas the unc-116(rf), ida-1::gfp and unc-116(rf); ida-1::gfp strains were short-lived.” => what is the difference between ‘unc-116(rf), ida-1::gfp’ and ‘unc-116(rf); ida-1::gfp’?
- “Strikingly, the ida-1::gfp transgenic…” = Strikingly, the ida-1::gfp transgenic animal…

Introduction
- “In addition to relying on standard diffusion…” => what is “standard diffusion”? Molecular motors are either processive on non-processive. There is a skating (dynein) or “biased diffusion” phenomenon but this needs to be explicitly explained (if applicable)
Response: This comes from our mathematician co-authors, since motion can be classified as standard diffusion or anomalous diffusion (sub- and super-diffusive). Because this is confusing, this wording has been removed.
- “While the klc-1 and -2 mutant worms had normal longevity” = While the klc-1 and -2 mutant worms revealed wildtype longevity
Response: This now reads: While the klc-1 and -2 mutant worms had the same lifespan as wild type…
- “… but combining ida-1::gfp with either klc mutation gave animals with a longer lifespan than wildtype worms or either parental strain” => this sentence needs a complete overhaul
Response: We have revised this text as follows, which we hope is easier to follow:
“While the klc-1 and -2 mutant worms had the same lifespan as wild type, the unc-116 mutant worms were short-lived. The expression of the ida-1::gfp transgenic animals also had a shorter lifespan. Unexpectedly, expressing ida-1::gfp in either the klc-1 or klc-2 mutant strains gave animals with a significantly longer lifespan than wild-type worms or the parental strains.”
Results
- “The ALA neuron is ideal for imaging, as it extends two axons down each side of the worm from the cell body in the head” => a pictographic representation of the ALA is recommended especially for the readers with less C. elegans expertise.
Response: Thanks for this suggestion. A diagram of the ALA neuron is now provided as fig. 1A.
- All strains need to better explained (defined). What is the resulting gene product of rh24sb79, km11 and ok2609? How large are the deletions? Are there frameshifts of premature stops? If knockout (ok2609) is there any literature evidence showing a Western to proof for knockout? If not authors need to do it.
Response: In the previous version the strains were described in the methods. We have now moved the most important information into the results. The klc-1 strain is truncated at amino acid 36, followed by QQTLFALRDEHEAATRILEANLISKTIYGMSTNKVSIRFRLIRTFFAQ-stop. It is therefore a functional null. The klc-2(km11) mutant is the only one available that lives past L1, and is complex (as described in the text). How much truncated protein is expressed is not clear, with two contradictory studies (Yang et al., 2005; Sure et al., 2018). Nevertheless, it is clearly a hypomorphic mutant, as shown in many studies.
- “Inappropriate branching of axons has been observed in zebrafish (Auer et al., 2015), but it has not been reported in C. elegans kinesin-1 mutants to our knowledge.” => The authors seem to make a point on the branching, thus it shall be quantified using existing image analysis tools.

© 2024. Published by The Company of Biologists under the terms of the Creative Commons Attribution License (https://creativecommons.org/licenses/by/4.0/).

Response: This has been scored by simple observation, and we have included this text: “with visual analysis identifying branching in 17/25 young adult worms.” We no longer emphasise this point in the paper.
- Page 5: “…we imaged single z-planes by spinning disk confocal microscopy” = we imaged single z-planes of ALA neurons by spinning disk confocal microscopy
Response: Corrected.
- Did the authors used levamisole to immobilize the worms? If yes, it needs to be mentioned because it has been reported to affect transport (Koushika lab).
Response: We apologise for not including this in the methods. We have now included the details and mentioned it in the results. Although it does affect transport, all worms have been treated in the same way and so the comparisons are valid.
- Page 6: “In addition, there were notable spots where particles clustered together and did not move, which likely correspond to the periodic swellings observed by EM along the length of the ALA neuron that contain clusters of DCV, some of which may occur at en passant synapses” => needs quantification
Response: We are not sure what quantification is requested here. However, as we are focussing on the movement of DCVs rather than the appearance of the clusters, we feel this is outside the scope of this study.
- Page 6: “When approaching such a cluster, the moving DCV could either stop or pass through. Clusters were sometimes seen to shed a DCV that then moved off in either direction.” => needs quantification
Response: The text has now been modified: When approaching such a cluster, the moving DCV could either stop or pass through. For anterograde DCVs, 75% moved through at least one cluster with 60 % stopping at a cluster (n=163 vesicles scored in 49 kymographs from 8 experiments) while ~100% of retrogradely transported DCVs moved through clusters and 22.3% stopped at least once (n=332 DCVs scored). Clusters could also shed DCVs that then moved off in either direction, with 48% of released DCVs moving anterogradely and 19% moving retrogradely.
- Table 1: Groups need to be compared using ANOVA to evaluate statistical differences.
Response: The existing results from the Komogorov-Smirnov test on the distribution of velocities is now included in table 1 rather than being in a separate table.
- “Comparison of the distribution of the individual segment velocities showed that both directions were significantly different to those in wild-type ida-1::gfp worms” => I don’t see any statistics.
Response: See comment above.
- “In contrast to the klc-2(rf) mutant, the complete loss of klc-1 had only a small, statistically insignificant effect on plus-end-directed mean velocity” => Once more no statistics comparing groups (t-test or ANOVA) are provided by the authors.
Response: See comment above.
- Page 7: “This clearly shows the greater distance travelled by dynein-driven DCVs within a set time in the klc-1(-) mutant compared to the wild-type and klc-2(rf) mutant.” => Run lengths values can be easily measured and blotted and proper statistic done (once more please refer to papers from established groups).
Response: See comments above. The K-S test is the best test to use for comparison of distributions of data, as in this case (Fig. 4). It is not appropriate to try to extract run lengths.
- Page 7: “swimming” => “thrashing assay” is the preferred term
Response: The studies in the literature that focus on worm locomotion use swimming rather than thrashing, as the motion is directional in the real world (e.g. Vidal-Gadea, A. et al. Caenorhabditis elegans selects distinct crawling and swimming gaits via dopamine and serotonin. Proc. Natl. Acad. Sci. 108, 17504–17509, 2011). We therefore prefer to use that term. However, we make it clear in the text that it is also called thrashing.
- Page 7: “indicating neuronal dysfunction” => Aldicarb assays is also a good assay to test for this
Response: Thank you for the suggestion. We have included aldicarb and levamisole assays (Fig. 5C, D: statistics in table S3), which proved to be very informative. We have also analysed effects on crawling (Fig. 5B, statistics in table S2) as well as swimming.
- Figure 5: once more proper statistics (such as ANOVA) is missing.
Response: T-tests were included as a supplementary table, but we have now used One-way ANOVA with Tukey-Kramer HSD test of the data from Fig. 5A and B. Full analysis is provided in tables S1 and S2. The aldicarb and levamisole data is analysed by a log-rank test (table S3). Key P-values are provided in the text where it is not too complicated to do so.
- Page 8: “the bending frequency of both unc-116(rf) and unc-116(rf); ida-1::gfp was extremely low and not significantly different” => it needs a t-test to underscore this

© 2024. Published by The Company of Biologists under the terms of the Creative Commons Attribution License (https://creativecommons.org/licenses/by/4.0/).

Response: See comment above.
- Page 8: “being somewhat worse than unc-116(rf) alone at later times” => scientific writing needs to be improved
Response: This has been changed.

Reviewer 2 Comments for the Author...
1. In Abstract, unc-116(rf), klc-2(rf) and klc-1(-). (rf) and (-) are not standard nomenclature. The authors need to clearly define them.
Response: This terminology has been removed from the abstract and is defined in the results section.
2. It is described that "Gaussian fitting revealed three peaks (Fig. S5), which likely correspond to kinesin-1 (slow), kinesin-3/UNC-104 (fast) and a mixture of the two (intermediate), as has been previously observed for mixtures of kinesin-1 and -3 motors". On the other hand, it is described that " Notably, however, the plus-end-directed velocities in the unc-116(rf) mutant, which still has wild type UNC-104 motors, is drastically slowed to a velocity not seen in normal axons, despite UNC-104 being a fast motor. This may be due to kinesin-3 motors detaching more readily from microtubules than kinesin-1 (Arpag et al., 2019; Arpag et al., 2014; Norris et al.,2014), particularly under load. The presence of the partially functional UNC-116 motor....." I cannot understand this argument. Please reconsider and rewrite them. If UNC-104 is faster, and the intermediate peak is generated by the cooperation between UNC-104(fast) and UNC-116(slow), unc-116(rf) should show faster movement.
Response: We have revised this text and hope it is now clear. The essential point is that UNC-104 may be particularly badly affected by any drag generated by partially functional kinesin-1 or dynein, and therefore can’t run at full speed. The drag may also cause transient detachments of UNC-104 from the microtubule, so reducing the velocity on the longer time-frame of imaging.
If slower movement in unc-116(rf) is caused by frequent detachment, the run length of DCB should be shorter in unc-116(rf). Is this correct?
This is an interesting point, but as it is not possible to measure wild-type run length in our assays because it is so continuous (see comments to reviewer 1) it would hard to be test this as requested.
A simple explanation is that UNC-104 is slower than UNC-116. Are there any publication that compared the velocity of UNC-104 and UNC-116?
Response: Yes, there is. The appropriate references are provided in the discussion.
"Alternatively, since dimerization of kinesin-3 motors has been shown to make them super-processive (Soppina et al., 2014), perhaps UNC-104 is unable to dimerise in the absence of functional UNC-116." There is no evidence. For instance, synaptic vesicles are transported by UNC-104 without UNC-116. Moreover, if this is case, the velocity of DCVs should not exhibit three peaks. The fastest peak should be generated by UNC-104 only.
Response: We agree that this was too speculative and have removed this text.
"Another possibility there is not enough UNC-104 present on the DCVs to be able to drive their rapid movement through the highly crowded axonal environment without the help of kinesin-1, as has been observed for DCV motility in MRC5 cells (Serra-Marques et al., 2020)." Again, presented evidence is not consistent with this scenario. If this is true, again, the velocity of DCV should not exhibit three peaks.
Response: We based this argument on the work of Serra-Marques et al., which was convincing to us. However, there is no evidence for this scenario, so we have removed this text.
3. The authors have suggested that UNC-116 is fundamental for the axonal transport of DCVs. Then, why is the axonal transport of DCVs almost diminished in unc-104 mutants? Could the authors quantify the motility of DCVs (velicity, run length etc.) in unc-104 mutants to show the UNC-116 dependent DCV movement only?
Response: This is a fascinating question, for which there is no clear answer. We discuss two possibilities in the text, and we hope our wording is now clear. Analysis of DCV motion in the ALA neuron in unc-104 mutants has been done to an extent by Zahn et al., who found that the fastest movements were lost without UNC-104. Rather than repeat this mutant analysis, our intention in the future is to use auxin-mediated degradation of kinesin-1 or kinesin-3 to see what happens to DCV movement without the complication of a mutant, partially functional motor restraining the DCV motion in ill-defined ways.
4. "Altogether, these data strongly suggests that kinesins-1 and -3 work in tandem to drive DCV along the ALA neuron". The word "tandem" is ambiguous. Does the authors use the word to say "cooperative" or "redundant"? Or does the authors use the word "tandem" to say that UNC-104 work in the exit from the cell body and UNC-116 work in the axon?

© 2024. Published by The Company of Biologists under the terms of the Creative Commons Attribution License (https://creativecommons.org/licenses/by/4.0/).

Response: We think the motors likely work together, but not redundantly, and that the latter possibility is the most plausible. We have removed the word 'tandem'.
5. Plus-end-directed movement was less processive and had an average velocity of 1.4 ± 0.68 ?m/s (Table 1).
(1) The word "processive" is used in enzymology to describe the behavior of enzymes, such as kinesin, that move along a substrate without detaching. While "processive" is used in the context of kinesin to represent "run length", its use to describe the run length of vesicles is not appropriate.
Response: Although this is commonly used with respect to vesicle movement (e.g. Serra-Marques et al., 2020, eLife, 9:e61302), we have removed this word.
(2) Please show the run length in Table S1.
Response: Please see previous comments explaining why run lengths cannot be measured for DCV movement in our movies.

Reviewer 3 Comments for the Author...
My main comment for improvement of the work is in presentation. For a conceptually straightforward paper, I thought that is overly long and somewhat heavy going. In particular, there is a large amount of discussion and citation of other literature in the results, that I think distracts from the author's findings and the clarity of their presentation. I would also suggest some attempt is made to condense the discussion to focus on the key findings here.
Response: We apologise for the problems with the text. We have moved much of the literature from the results, making it easier to follow, and have also streamlined the text throughout. We agree that the discussion was too long and unfocussed. We have rewritten it substantially, with the new data from the aldicarb and levamisole experiments making it much easier to discuss the context of our results.

Second decision letter

MS ID#: JOCES/2024/262148

MS TITLE: The role of Kinesin-1 in neuronal dense core vesicle transport, locomotion and lifespan regulation in C. elegans

AUTHORS: Anna Gavrilova, Astrid Boström, Nickolay Korabel, Gino B Poulin, Sergei Fedotov, and Victoria J Allan

I am happy to tell you that your manuscript has been accepted for publication in the Journal of Cell Science, pending standard publication integrity checks.

© 2024. Published by The Company of Biologists under the terms of the Creative Commons Attribution License (https://creativecommons.org/licenses/by/4.0/).
